# Racism in healthcare: a scoping review

**DOI:** 10.1186/s12889-022-13122-y

**Published:** 2022-05-16

**Authors:** Sarah Hamed, Hannah Bradby, Beth Maina Ahlberg, Suruchi Thapar-Björkert

**Affiliations:** 1 Department of Sociology, Uppsala University, Uppsala, Sweden; 2 Skaraborg Institute for Research and Development, Skövde, Sweden; 3 Department of Government, Uppsala University, Uppsala, Sweden

**Keywords:** Racism, Discrimination, Healthcare, Review

## Abstract

**Background:**

Racism constitutes a barrier towards achieving equitable healthcare as documented in research showing unequal processes of delivering, accessing, and receiving healthcare across countries and healthcare indicators. This review summarizes studies examining how racism is discussed and produced in the process of delivering, accessing and receiving healthcare across various national contexts.

**Method:**

The PRISMA guidelines for scoping reviews were followed and databases were searched for peer reviewed empirical articles in English across national contexts. No starting date limitation was applied for this review. The end date was December 1, 2020. The review scoped 213 articles. The results were summarized, coded and thematically categorized in regards to the aim.

**Results:**

The review yielded the following categories: healthcare users’ experiences of racism in healthcare; healthcare staff’s experiences of racism; healthcare staff’s racial attitudes and beliefs; effects of racism in healthcare on various treatment choices; healthcare staff’s reflections on racism in healthcare and; antiracist training in healthcare. Racialized minorities experience inadequate healthcare and being dismissed in healthcare interactions. Experiences of racism are associated with lack of trust and delay in seeking healthcare. Racialized minority healthcare staff experience racism in their workplace from healthcare users and colleagues and lack of organizational support in managing racism. Research on healthcare staff’s racial attitudes and beliefs demonstrate a range of negative stereotypes regarding racialized minority healthcare users who are viewed as difficult. Research on implicit racial bias illustrates that healthcare staff exhibit racial bias in favor of majority group. Healthcare staff’s racial bias may influence medical decisions negatively. Studies examining healthcare staff’s reflections on racism and antiracist training show that healthcare staff tend to construct healthcare as impartial and that healthcare staff do not readily discuss racism in their workplace.

**Conclusions:**

The USA dominates the research. It is imperative that research covers other geo-political contexts. Research on racism in healthcare is mainly descriptive, atheoretical, uses racial categories uncritically and tends to ignore racialization processes making it difficult to conceptualize racism. Sociological research on racism could inform research on racism as it theoretically explains racism’s structural embeddedness, which could aid in tackling racism to provide good quality care.

**Supplementary Information:**

The online version contains supplementary material available at 10.1186/s12889-022-13122-y.

## Background

This scoping review summarizes studies that look at how racism is discussed and produced in the process of delivering, accessing and receiving healthcare. Racism can be defined as a form of social formation embedded within a network of social, economic, and political entities in which groups of people are categorized and hierarchically ordered through a historical process of racialization [1]. Groups of people who are racialized as inferior, henceforth referred to as racialized minorities, are devalued, disempowered, and subjected to differential treatment in various institutions, including healthcare, resulting in negative material consequences affecting people’s living conditions, everyday lives, including access to healthcare and health outcomes [2]. We use the term minority herein to indicate groups of people who are minoritized as they are subjected to unequal power relations. Racism is a dynamic historical process that continuously undergoes change and finds new forms of political, social, cultural, or linguistic expressions [3]. In contrast to the official, recognized institutionalized racism that existed in most Western settings prior to the Second World War, contemporary racism persists through more normalized covert or invisible processes rather than explicit expressions of racism [4]. These processes operate at multiple interrelated levels, ranging from the individual to the structural within existing structures [5].

In healthcare, as in other institutions, racism continues to persist and constitutes a major barrier towards achieving equitable and responsive healthcare. This is documented by research showing differential and unequal processes of delivering, accessing, and receiving healthcare across various countries and healthcare indicators [2, 6] including diabetes care [7], mental healthcare [8], maternal healthcare [9], preventive vaccination [10], end-of-life care [11], cardiology care [12] and pain management [13]. Research has also documented that racialized minorities not only receive inadequate quality healthcare but are also viewed as less desirable healthcare users compared to majority groups [14]. A systematic scoping review of studies looking at healthcare users’ perspectives on racism in healthcare shows that racialized minority healthcare users are alienated due to racism and lack of empathy resulting in inadequate healthcare [15]. A meta-analysis [16] also shows that healthcare users who experience racism have higher odds of reporting lower trust in healthcare, lower satisfaction with care, and perceived quality of care. Meta-analysis reviews [17–19], as well as a scoping review of both qualitative and quantitative studies [20], show that healthcare staff produce racism unconsciously as they exhibit implicit racial bias, i.e., negative attitudes and stereotypes against racialized minorities relative to majority groups within the context of healthcare.

Although the volume of scientific research on the various ways racism affects healthcare has grown steadily [2], racism and its damaging effect on the livelihood and health of racialized minorities [21–23] persists and consequently constitute an injustice that needs to be addressed. Some systematic meta-analyses and scoping reviews regarding various dimensions of racism in healthcare have been conducted. These have examined evidence involving healthcare staff’s implicit racial bias [17–20], antiracist interventions in healthcare [24], healthcare users’ utilization of care [16], public health understanding of racism in healthcare [25], as well as other topics [26]. The importance of these reviews notwithstanding, these reviews focus on specific dimensions pertaining to racism in healthcare and hence do not examine the full extent of the existing evidence on racism in healthcare. We argue that to understand how racism is produced in healthcare, given that racism is a complex social formation that is embedded in structures of modern societies, a full overview of the various operative dimensions of racism is needed. Put in other words, focusing on racism as the object of research instead of specific topics, offers an in-depth understanding of the complex nature of racism that is not amenable to a more health topic specific review. To our knowledge, there have not been any reviews that have examined all empirical evidence on the topic of racism in healthcare. Conducting such a review is important in order to incorporate the growing number of articles on persistence of racism in healthcare. This calls for a description of the content of the studies in order to a) gain an overall comprehensive insight into what has been conducted regarding the various dimensions of racism in healthcare; b) through acquiring an overall picture of the research, identify existing knowledge gaps in the research that might aid researchers in explaining what further research is needed that can explain why racism continues to persist in healthcare. Since the topic of racism in healthcare extends over several disciplines and research methodologies [2], and in order to capture an overview of both the qualitative and quantitative research, we conducted a scoping review. Scoping reviews describe the characteristics of research, scope a body of literature, especially when a body of literature has not been comprehensively reviewed or when the literature is scattered and heterogeneous [27] as in the case of the topic herein. Noteworthy, is that we do not aim in this review to evaluate the strength of evidence of the reviewed articles, nor do we aim to evaluate the methodological rigor of the reviewed articles as is usually the case in meta-analyses. Rather, as delineated, the aim here is to describe the content of the research available on racism in healthcare and to identify existing knowledge gaps.

Before the material and methods of this scoping review are presented, a short note on terminologies used in this review is warranted. As this review includes articles from various national contexts, variations in what constitutes healthcare in these contexts exist. Therefore, for the purpose of this review, healthcare is defined as including health and dental care including medical and dental educational settings and elderly care but excludes military veteran care. Consequently healthcare staff are defined as a broad range of staff including nurses, dentists, midwives, physicians as well as other stakeholders related to the field of healthcare such as nursing aids and care givers. Healthcare users in this review are defined as all individuals who used the healthcare system as per the definition provided i.e., including medical and dental care. The review thus examines articles that look at how racism is discussed and produced in the process of delivering, accessing and receiving healthcare and not articles that look at the effects of racism on health outcomes or health status. The review therefore, aims to map all articles that focus on how racism is discussed and produced in healthcare including differential treatment due to racism, how racism affects healthcare interactions, experiences of racism and antiracist interventions as well as other aspects revealed by the review.

A wide variety of definitions exist in the literature concerning how to describe groups of people who are subjected to racism depending on the national contexts and on whether racial categories exist as legal categories in a given context. As an example, in the USA racial categories such as Black and White exist as legal categories while in many other European contexts, these categories are illegal [28]. In this review, and building on arguments made by Fanon [29] and Miles [30] on the importance of focusing on the process of racialization rather than ‘race’, the terms racialized minorities and majority groups are used to describe healthcare staff and users belonging to dominated and dominant groups. We use the term racialized minority to specify groups of people who are numerically a minority but who are also racialized as inferior in comparison to the majority groups and are thus subject to differential and unequal power relations and treatment in various institutional contexts. Although, we use the term racialized minority and majority in this review, the reviewed articles used a variety of different terminologies to indicate different ethnic groups. When results are reported from the reviewed articles in the Result section of this scoping review, we will use the terms that are used in the reviewed articles. Terms such as Black and White which indicate racial categories produced by historical racialization processes will be capitalized to indicate that they are social constructs. These terms are viewed as social constructs and they will be used if they are cited in the articles being reviewed when describing results. The use of these racial categories in the reviewed articles will be problematized in the Discussion section.

## Materials and methods

A scoping review has been conducted to examine the empirical qualitative, quantitative and mixed method studies on racism in healthcare and to identify any research and knowledge gaps. For this scoping review, we followed the published PRISMA (Preferred Reporting Items for Systematic reviews and Meta-Analysis) extension for scoping reviews (PRISMA-ScR) [31] to define the context and the concepts, to search, formulate inclusion criteria, extract and chart data in flow charts.

This scoping review aims to answer the following research questions:

1. *What is the content of studies that examine racism in healthcare? In other words, what do the studies tell us about racism in healthcare*, *including all aspects of racism in healthcare, as described in the introduction?*
2. *What are the existing research and knowledge gaps that current studies do not address in relation to racism in healthcare that may be useful in a) understanding how racism is produced in healthcare and b) how racism persists in healthcare.*

### Search strategy

Following the PRSIMA methodology, the search was conducted according to the following timeframe, databases and search terms:

- *Time frame*: No starting date limitation was applied for this review but the end date was December 1, 2020 to allow the authors enough time to review the selected articles.
- *Data bases*: the following databases and electronic journal collections were searched for English language studies: PubMed, PsycInfo, ASSIA and Scopus. These databases were chosen for being relevant for healthcare research (PubMed and PsycInfo); or multidisciplinary broad databases (Scopus) that cover all subject areas and are keywords databases; or databases that cover research on racism in healthcare in sociological research (ASSIA).
- *Search terms*: The following search terms were used: Topic (racism* or racial* or racialization* or racialisation* or “racial bias” or discrimination* or “structural racism” or “structural discrimination” or “institutional discrimination” or “institutional racism” AND TOPIC: “healthcare”, “dental care”, “healthcare staff”, “healthcare professionals”, “healthcare providers”, “healthcare users”, “patients”. The choice of search terms used in this scoping review was conducted in consultation with an acadmic librarian specializing in public health research. After consultation with the librarian terms such as stereotypes, prejudice, culture and ethnicity were not included as search terms in this review as these terms do not necessarily reflect racism in healthcare and could result from other practices. Additionally, this scoping review is broad and including even broader search terms would result in a magnitude of results that would not necessarily have to do with racism so a decision was made by the authors to limit the search terms of this review. As this might have limited some results, this limitation will be presented in the discussion section.

### Inclusion and exclusion criteria

Research papers in languages other than English were excluded. Only peer reviewed articles with empirical data were included. All literature reviews, conceptual and theoretical articles were excluded and no grey literature was included in this review. Studies of any design (qualitative, quantitative as well as mixed method studies i.e., studies that use both qualitative and quantitative methodology) that looked at racism in healthcare as per the explanation provided in the aim and definition of what constitutes racism in healthcare were included. All articles concerning the effects of racism on health status, racial health disparities and differential health outcomes were excluded from this study as this review is on racism in healthcare and not racial differences in health outcomes.

### Data extraction and coding

**Fig. 1:**
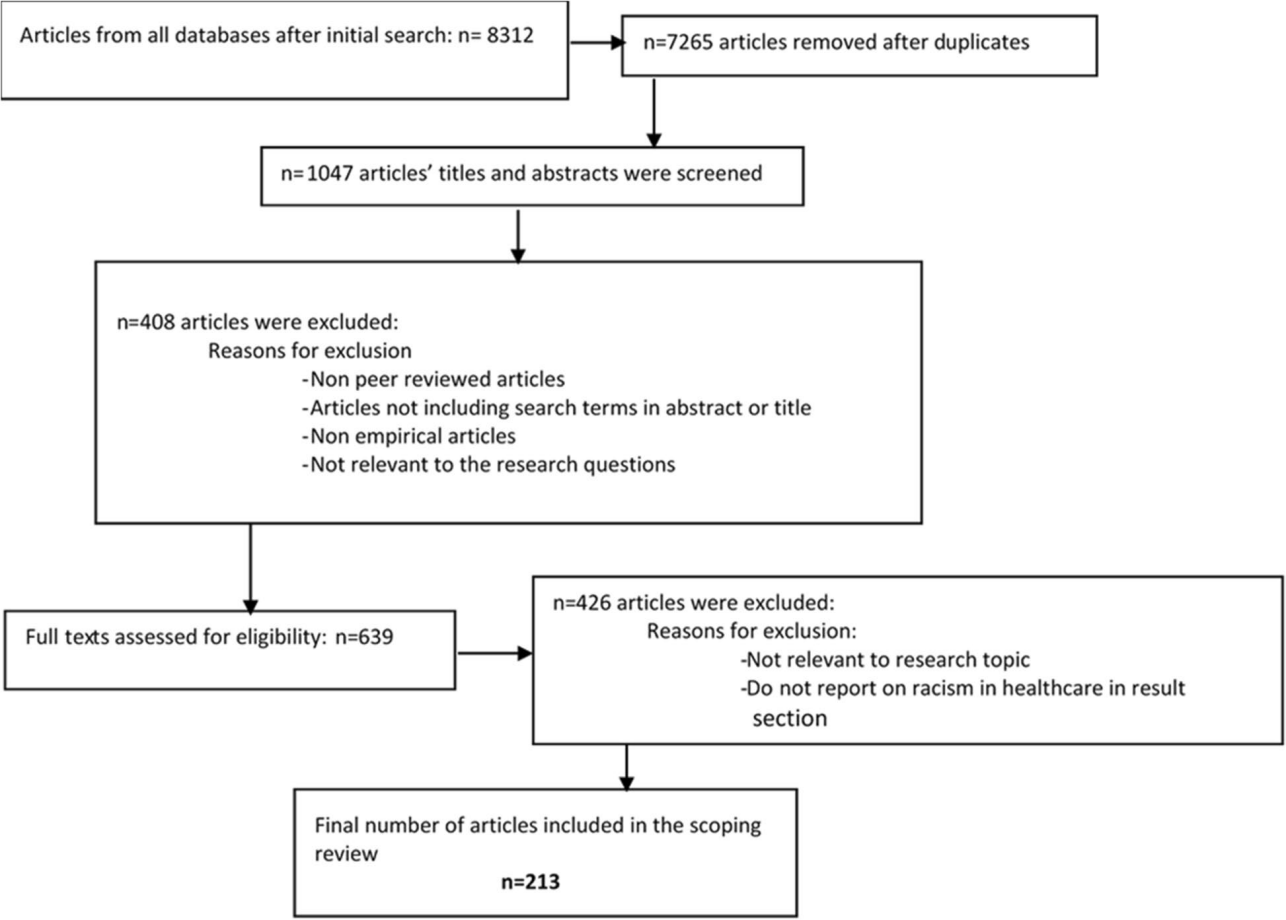
PRISMA diagram of the study selection process

The initial search was conducted using the selected search terms in the chosen databases. When the initial search was conducted, the search was limited to the selected date but also to English language, peer empirical reviewed articles as well as to articles that had full texts provided by our university library. As an example, using the search terms with its various iterations in the database ASSIA, generated 3149 initial hits (English peer reviewed articles with full texts). All the initial search hits from all databases were then collected in one file. The total number of articles from all databases after the initial search was 8312. Excel 2016 spreadsheet and Zotero software were used to store, organize and code the data. Duplicates were then removed and all remaining articles’ titles and abstracts were reviewed. The results are shown in the PRISMA flow chart in Fig. 1.

Two reviewers screened all titles and abstracts to assess eligibility for inclusion. Articles were excluded based on the exclusion and inclusion criteria (remaining *n* = 639). All articles which included the search terms in their title and/or abstract were included in this stage. If it was unclear whether the article should be included or in case an abstract did not exist, the decision was made to include or exclude after reading the full article in question and after a discussion with all reviewers was conducted.

In the next stage, the full text of all of the articles that were selected were read and screened by two reviewers. Once the final set of articles were selected based on their relevance to the research question, articles were coded and categorized using Excel 2016 (remaining *n* = 213). All reviewers were involved in the coding process (see Fig. 1 and the PRSIMA checklist in additional file 1).

**Fig. 2:**
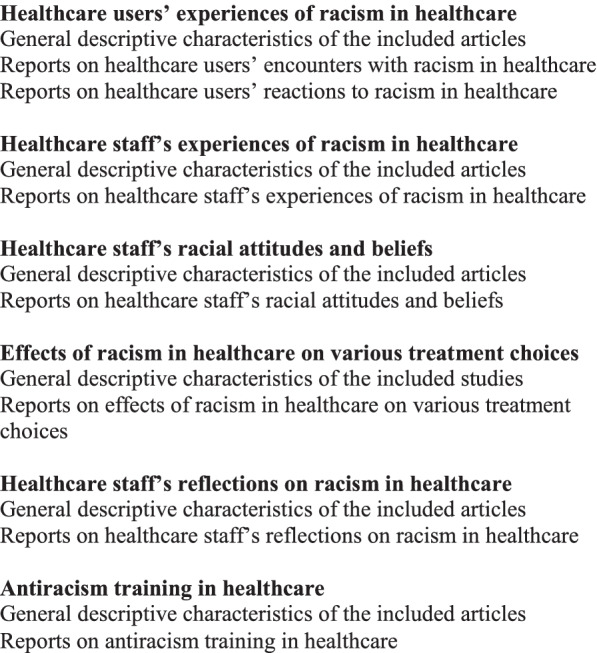
Diagram of categories and sub-categories of the result section

Data were summarized by year of publication, geographical location, aim of article, methods and key findings in relation to racism in healthcare. These summaries of all 213 articles are illustrated in additional file 2. If the method was not clearly stated in the reviewed article, this was reported in the table as unclear method section. However, this review will not look further into the methodology of the articles as it does not fall into the aim of the review. Since the aim of this scoping review is to give an overview of the content of the reviewed studies in relation to the various dimensions of racism in healthcare, the data were coded based on the key findings in relation to the topic herein. All authors discussed the coding of the data and consequently it was decided that the data would be classified into the categories illustrated in Fig. 2 below:

## Results

Given that this review reports on findings from 213 articles, it is not possible to set out all the results of every article included. However, additional file 2 gives a summary of the main results in relation to racism in healthcare in all 213 articles that were scoped. The result sections starts with a short description of the main, general findings of the articles. This will be followed by an illustration of the categories and subcategories as demonstrated in Fig. 2.

### Descriptive findings: characteristics of articles included

**Fig. 3:**
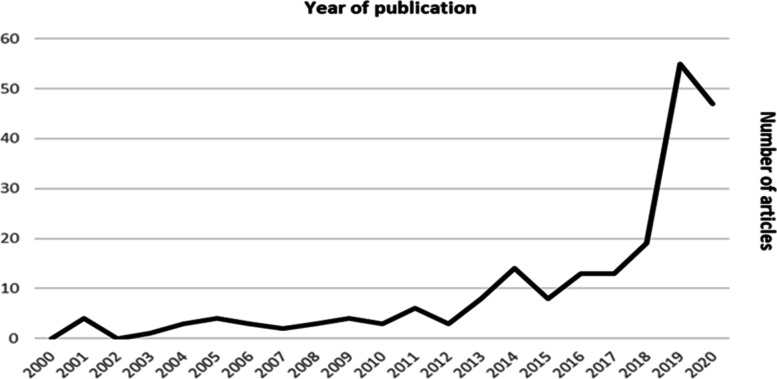
Number of empirical articles on racism in healthcare published per year

The oldest article included in the review was from 2001, which means that no article (found through the search process of this review and which met the inclusion criteria) was published prior to that date. From 2005, an increase in the number of published articles on racism is observed (Fig. 3), which indicates an increased academic interest in the subject. A sharp increase in the number of articles published per year was observed in 2019 and 2020 with an increase of 65% from 2018 to 2019.

Some of the general characteristics of the reviewed articles are described in Table 1 below including type of methodlogy, focus on racism and country of research.

**Table 1:** General characteristics of the reviewed articles

**Article characteristics**	**Number of articles (ca. %) **
Quantitative articles	90 (42%)
Qualitative articles	109 (51%)
Mixed articles	14 (6.6%)
Articles focusing mainly on racism*	135 (63%)
Articles examining only healthcare users	114 (54%)
Articles examining only healthcare staff	71 (33%)
Articles evaluating antiracism training/workshop for healthcare staff and or students	14 (6.6%)
Geographical location*	
*USA *	*142 (67%)*
*UK*	*15 (7%)*
*Canada*	*15 (7%)*
*Australia*	*11 (5%)*
*New Zealand*	*6 (3%)*
*Israel*	*5 (2%)*
*Sweden*	*3 (1.4%)*
*France*	*3 (1.4%)*
*Belgium *	*3 (1.4%)*
*Spain *	*3 (1.4%)*
*Finland*	*2 (0.9%)*
*Brazil*	*2 (0.9%)*
*Portugal*	*1 (0.5%)*
*Germany *	*1 (0.5%)*
*Slovakia*	*1 (0.5%)*
*Ireland*	*1 (0.5%)*
*Romania*	*1 (0.5%)*
*Turkey*	*1 (0.5%)*
*India*	*1 (0.5%)*
*Malaysia*	*1 (0.5%)*
*South Africa*	*1 (0.5%)*

*These articles’ main research question was to explore aspects of racism in healthcare. This means that 78 (37 %) of the reviewed articles have other research questions i.e., other than racism in healthcare, but nonetheless report on racism in healthcare in the abstract and in their results and so were included in this review

*Please note that there are articles that are multinational articles where research has been conducted in multiple countries

**Fig. 4:**
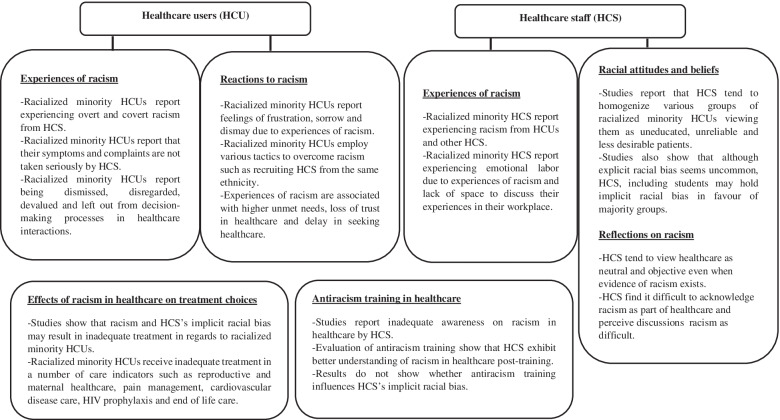
Summary diagram of the various categories of the result section

Below we illustrate the various categories and subcategories that resulted from our coding. Under each category descriptive general characteristics of each category is illustrated, followed by a description of the important content of the included article. Noteworthy, is that some articles are included in more than one category. Moroever, since this review has scoped a large number of articles, generating a lengthy result section, a summary diagram of the important results from the various categories is included in Fig. 4 below.

### Healthcare users’ experiences of racism in healthcare

This section describes the content of articles that report on experiences of racialized minority healthcare users’ experiences of racism in healthcare including both their perceived racism in healthcare encounters as well as their reactions to racism. The section starts by giving some general descriptive characteristics of these studies followed by an overview of their important content.

#### Descriptive findings: characteristics of articles included

Of the 213 articles in this scoping review, 117 articles examine healthcare users’ experiences of racism in healthcare. Of these 69 are qualitative studies, 41 are quantitative and 7 use mixed methods. These include articles that have racism as their main focus as well as articles that do not have racism as their main focus and only report on racism in their abstract and result section.

Most of the articles that have racism as their main focus report on healthcare users’ experiences of racism in healthcare in general in a variety of contexts i.e., these articles do not look at specific healthcare settings or experiences related to specific treatments or illnesses. These include both qualitative articles [32–40] and quantitative studies [10, 41–62] and two mixed method studies [50, 63, 64].

Various national contexts are represented including the USA [32, 33, 37–41, 43, 46–48, 52, 54, 58, 59, 65]. Other articles are from Brazil [34], multi-European settings [36], Mexico [35], New Zealand [49], Canada [51] and France [57]. All the articles from outside the USA are qualitative articles except for the article from France.

Other articles examine experiences of racism within specific illnesses or healthcare contexts. These include qualitative articles looking at hypertension care in the USA [66], decision making processes in diabetes care in the USA [67], depression care in USA [68], HIV-care in USA [69–72], mental care in USA [73], cardiovascular disease care in USA [74], reproductive, maternal and perinatal healthcare in USA [75–78], and chronic renal failure care in Brazil [79]. Quantitative articles examine how experiences of racism in HIV-care [80, 81], sickle cell disease management in the USA [82] mental healthcare in UK [83] and in USA [8, 84], diabetes care in USA [85–89], dental care in the USA [90, 91] and maternal care in USA [92]. Two studies employ a mixed methodology and look at mental care [93], pain management [94], and heart failure therapy; all in USA [95].

Some of the articles that do not have racism as their main focus set out to examine experiences, perceptions, facilitators and barriers of racialized minority healthcare users’ with healthcare and healthcare encounters in various contexts, mostly outside of the USA. These include reports of racism in healthcare encounters in Canada [96–101], Australia [64, 102], the UK [103, 104], USA [105–108], India [109], Belgium [110], Malaysia [111], Spain [112] and Israel [113]. All of these articles use qualitative method except one article from the USA [105], and one mixed method article also from the USA [107].

Articles that do not have racism as their main focus and look at specific healthcare contexts or indicators examine the following: trust in healthcare in the USA [114], trust in physicians in the USA [115], experiences of chronic illness in Australia [116], burn injury in pediatric care in Australia [117], pediatric acute care in Canada [118], acute care in Australia [119], end of life care in the USA [120], mental healthcare [121] in the USA [122], reproductive, maternal and perinatal care in the USA [123–128], in Australia [129] and Canada [130], cancer care in the USA [131–133] and the UK [134], dental care in the USA [135], diabetes care in the USA [7, 136], sexual healthcare in the USA [137], and finally perceptions of patient centered care in the USA [138]. The majority of these articles are qualitative except for 5 articles [7, 114, 115, 129, 133, 139] as well as one mixed method article [121].

#### Racialized minority healthcare users examined in the reviewed articles

Studies focusing on specific racialized minorities examine mostly experiences of African Americans/Blacks in USA (*n* = 28) [7, 32, 33, 38, 39, 43, 45, 53, 55, 65, 67, 69, 71, 72, 75, 77, 78, 81–83, 93, 115, 120, 122, 127, 131, 140, 141]. Other studies in the USA look at Hispanics (*n* = 10), [47, 52, 54, 107, 108, 121, 135–138], American Indians [48, 85, 106], as well as other ethnicities such as Asian Americans [54, 114].

Other studies look at experiences of Indigenous people outside of USA (*n* = 16) [35, 51, 64, 64, 78, 96–102, 117, 118, 129, 130], Pakistani Muslims [103] and Sub-Saharan Africans [112, 113]. Some studies do not specify a minority category and use terms such as People of Color [37, 123–126], migrants/refugees [36, 57, 104, 110] and minorities [56, 58, 63].

#### Report of healthcare users’ encounters with racism in healthcare

Quantitative studies looking at reports of racism in healthcare that include both majority and racialized minorities (mostly African Americans) show that racialized minorities are more likely to report experiences of perceived racism and inadequate healthcare, compared to majority groups in various contexts such as in the USA [10, 46, 52, 58, 59, 80, 87, 142], New Zealand [49], and France [57]. These experiences of discrimination are reported in connection to mental healthcare in the USA [8].

Racialized minority healthcare users report being subjected to both overt and covert racism in healthcare interactions from healthcare staff in a number of studies. For example, qualitative studies from USA report that African American women report the use of racial slurs by healthcare staff [99, 127] as do Aboriginal women in Canada [101]. Racialised minority healthcare users also report being reprimanded in a way that felt unjustified and scolded by healthcare staff such as reported by African American women in the USA [33]. One study in the UK [143] also reports that experiences of racism in healthcare by racialized minorities (Pakistani community) is seen as a disruption of the principles of solidarity and equity of healthcare.

The reviewed articles report that racism is experienced in different ways. These include negative assumptions from healthcare staff towards racialized minority healthcare users. As an example qualitative studies show that African American healthcare users report that they perceive experiences of racism as avoidance of touch by healthcare staff [141], exclusion from decision making processes in healthcare interactions [7, 39, 67] as well as lack of respect [135]. Being scolded, treated rudely and with apathy by healthcare staff is reported in a number of qualitative studies e.g. by Hispanics and Latinas in USA [137, 138] and women of color also in USA [126]. Moreover, racialized minority healthcare users in other contexts outside of USA also share the same perceptions of being left out of decision making processes as well as being dismissed and discarded. For instance qualitative studies looking at people of non-European migrants in Sweden, Portugal and Germany [36] as well as in Belgium [110] report that these healthcare users feel that their symptoms and complaints are not taken seriously by healthcare staff. First Nation and Indigenous healthcare users in Canada [96, 100] and Indigenous people in Kerala India [109] also report being trivialized in healthcare encounters as demonstrated in qualitative studies.

Some of the qualitative studies that explore experiences of e.g. African Americans [40, 122, 128] and Aboriginals in Canada [101] also report that these groups perceive racism as institutional and connected to historical racism of their respective countries. Racism in healthcare is described as connected to the historical trajectories of colonialism, and connected to the historical struggle of minorities against racism and colonial structures [40, 101, 128]. Racism is moreover viewed as a common experience throughout their everyday interactions both in healthcare and outside [122].

#### Reports on healthcare users’ reactions to racism in healthcare

Qualitative studies show that experiences of racism by various racialized minority healthcare users are linked with feelings of frustration, sorrow, dismay and feeling insignificant as reported by e.g. Aboriginals in Australia [116] and African Americans [33, 68]. The qualitative studies also show that racialized minority healthcare users avoid seeking healthcare due to experiences of racism in healthcare services. For instance, in Spain and Brazil, Sub-Saharan African healthcare users and various other racialized minorities similarly report being afraid of the healthcare system due to experiences of racism in healthcare [34, 112] which may lead to healthcare users  preferring healthcare staff of the same ethnicity as themselves [76, 123]. Preference for healthcare staff of the same ethnicity as a result of experiences of racism by racialized minorities was also shown in a quantitative study from the USA [53].

As racialized healthcare users emphasize the role of trust and equality in healthcare [71, 134, 140], mistrust in healthcare staff [66, 115] and healthcare [69, 120] is a common reaction from healthcare users who experience racism. Quantitative studies from USA show that perceived racism is inversely associated with trust in healthcare staff and positively related to distrust in healthcare [45, 55, 114]. Quantitative studies also show that racism in healthcare experienced by racialized minorities is associated with higher levels of unmet needs, lower satisfaction with care as shown by a study from New Zealand [49] and another from Canada [51]. Additionally, the experience of racism in healthcare is associated with lower medication adherence in mental health illness in UK [83], increased likelihood of delaying both cholesterol screening visits [55] and dental exams in USA [91]. Moreover, other studies from USA show that the experience of racism in healthcare is also associated with an increased number of teeth lost among healthcare users [90], worse diabetes care [89] as well as avoiding initiating the process of kidney transplant evaluation [139]. However, perceived racism in healthcare in the USA, as shown in two study [85, 87] is not associated with lower frequency of the uptake of services for diabetes [85] and of diabetes medications for hyperglycemia [87].

Various coping mechanisms used by healthcare users were reported in the reviewed articles. Qualitative studies show that in USA, African American women use religious faith and the church to deal with racism in healthcare [120]. African American healthcare users also report acting assertively [33, 39] and employing respectability tactics (making providers aware of their social status, educational background etc.) to convince healthcare staff to take them seriously [39, 144]. African American women also describe conducting research on their illness as well as dressing well for consultations, taking initiatives and asking healthcare staff questions so as to be perceived as knowledgeable [115]. The importance of family and peer support is also reported as a way of coping with racism in healthcare. Other strategies such as recruiting healthcare staff from the same ethnicity is discussed by African Americans [116] as well as People of Color in general in USA [37].

### Healthcare staff’s experiences of racism in healthcare

Twenty-seven articles explore racialized minority healthcare staff’s experience of racism in their workplace [50, 145–170]. Most of these article set out to examine healthcare staff’s experiences of racism, except for two articles that look at healthcare staff’s mental health issues in the workplace in USA [147], career progression [150, 151] and quality of healthcare provided to migrant elderly care residents [171].

All these studies were qualitative studies except for 4 quantitative studies [150, 154, 164, 168] and one mixed study [166]. Of these studies 7 studies explore experiences of racialized minority healthcare staff in USA [147–150, 152, 158, 161, 162, 165, 166, 168–170]. Other studies look at experiences of racialized minority healthcare staff in various national contexts including UK [145, 159, 160, 167], New Zealand [152, 164, 171], Canada [153, 163], Israel [50, 156], Finland [154, 168] and Sweden [155].

#### Racialized minority healthcare staff examined in the reviewed articles

These articles looked at a variety of minority groups. These included as reported by the articles, Blacks/African Americans in the USA [147, 148, 165, 169, 170], Blacks in UK [160] and Canada [163], overseas and immigrant healthcare staff in the UK [145, 159, 167], in Canada [153], Finland [154, 167] and Sweden [155]. Healthcare staff of Color are examined in the USA [146, 149, 166] as well as Hispanic in the USA [162], Filipina in the USA [161], Arabs in Israel [156, 172], Maori and Asian Pacific in New Zealand [164] and a combination of various racialized minorities  in New Zealand [152] and the USA [151].

#### Categories of healthcare staff examined in the reviewed articles

The reviewed articles examine different categories of healthcare staff. However, most examine nurses’ experiences of racism in healthcare [145, 149, 152, 153, 159–162, 168, 169]. Others examine both physicians and nurses [50, 156, 165, 170], physicians [154, 163, 166], medical settings’ members [147, 148], care workers [155, 158, 164, 167, 171], and oral maxillofacial surgeons [150].

#### Reports of healthcare staff’s experiences of racism in healthcare

Racialized minority healthcare staff describe incidents of racism by other staff members [150, 154, 162, 167, 170]. As an example, a study of immigrant physicians from Finland [154] and another of immigrant nurses from the UK [167] demonstrate that healthcare staff report being bullied by other healthcare staff in the workplace. Another study of African American nurses in the USA, report that these nurses demonstrate feelings of mistrust towards other healthcare staff due to experiences of racism [169] and discuss experiencing both overt and covert racism in the workplace. In Israel, blatant racism is experienced by nurses from healthcare users who refuse to be treated by Arab nurses with reports of both verbal and physical violence from healthcare users [156].

Nurses of color in the USA report undertaking excessive emotional labor and experiencing depletion due to racism from healthcare users [149]. Navigating racism in medical education in the USA is also reported to entail immense emotional labor [147]. In medical educational settings, medical students of racialized minorities in the UK report difficulties in navigating racism, which had an effect on their mental health. Feelings of racism in healthcare led to stress and mental health issues as reported by a study of immigrant nurses in Canada [153]. One study conducted in both the USA and New Zealand, report that racialized minority nurses suppress their ethnic identities in order to navigate their workplace due to lack of awareness by other nurses on issues of racism [152].

One study from Canada shows that when complaining about issues of racism to managers, nurses are framed as trouble-makers and their complaints are dismissed [153]. Lack of support from managers is also described in the USA by care workers [158] and in one case, majority nurses in the USA and New Zealand report they were punished for supporting racialized minority nurses who encountered racism [152]. As a consequence of the difficulties in reporting racism, some studies report that healthcare staff have to tolerate racism from healthcare users. As an example a study from the USA looking at Hispanic nurses experiences of racism [162], a study from New Zealand of migrant nurses [171] and a study of immigrant care workers in Sweden [155] report that these healthcare staff are expected to tolerate and absorb racism from healthcare users as the latter are sick and vulnerable. Moreover, Black nurses in the USA report having lost confidence in their abilities [160] since they are told that they are not good enough for their job. Experiences of exclusion from clinical teams and loss of job opportunities is also reported by overseas nurses in the USA [145].

### Healthcare staff’s racial attitudes and beliefs

This category includes 33 articles, made up of 12 qualitative, 20 quantitative and one mixed method studies. A number of qualitative articles in various national contexts explore healthcare staff racial attitudes and beliefs. These include qualitative analyses that examine healthcare staff’s beliefs and perceptions in regards to various racialized minorities as well as quantitative analyses that examine healthcare staff’s implicit racial bias.

Qualitative articles that examine beliefs and perceptions of healthcare staff in regards to racialized minorities are all articles that have not primarily set out to examine racism. These articles mainly explore how healthcare staff perceive interactions with Roma people in Slovakia [173], Spain [174], Romania [175] and in multi-European contexts [176]. Moreover, these studies look at American Indians in the USA [106], African American women in the USA [177], Micronesian women in Hawai ‘i in the USA [178], Indigenous people in Australia [179], Asians in the UK [180, 181] as well as racialized minorities in general in Belgium [182], in Ireland [183] and in France [14].

A number of quantitative articles describe healthcare staff’s racial bias in healthcare encounters [61, 184–202]. All of these articles are conducted in the USA except for one article from France [197], and two from New Zealand [187, 192]. A variety of categories of healthcare staff are examined in these articles including surgeons [191], pediatrians [195, 196], physicians [61, 188, 189, 193, 198, 200], medical students [185–187, 192], nurses [191], and various other categories of staff [184, 197, 199, 202].

#### Reports of healthcare staff’s racial attitudes and beliefs

Qualitative studies suggest there is a tendency to homogenize racialized minorities [174, 181, 183]. In the USA, Black men are judged as uneducated and less reliable by both White and Black healthcare staff [13]. In the UK, a study found that majority healthcare staff tend to homogenize racialized minority healthcare users, view Asian minorities as holding irrational religious beliefs, while at the same time denying the existence of racism [181]. In a study in Spain, healthcare staff have been shown to have negative perceptions towards Roma women subjected to intimate partner violence [174]. Additionally, in Belgium and France healthcare users belonging to racialized minorities, especially Muslims, are seen as problematic and frustrating [14, 182]. A study in Ireland shows that healthcare staff perceive healthcare users from racialized minorities in maternal care as demanding, too emotional and dramatic [183].

Quantitative studies show that healthcare staff from the USA have implicit racial bias in favor of Whites even when they primarily state that they do not have such bias (e.g. Haider et al., 2014, 2015) [190, 191]. A study in the USA shows that implicit racial bias favoring Whites increase at the emergency department when the department is overcrowded with healthcare users [194]. Yet another study from the USA shows that implicit racial bias increases with increased healthcare staff burnout [188]. Another study from the USA showed implicit racial bias in favor of Whites healthcare users, both adults and children [195], while another study, also from the USA shows that female and African American physicians exhibit less implicit racial bias than men and White physicians [200]. Studies from New Zealand also show that medical students exhibit implicit racial bias in favor of Europeans and against Maori groups [187, 192]. Two studies in this review found that French physicians do not exhibit implicit racial bias [197] and pediatrician residents in the USA exhibit weaker implicit bias in comparison to existing findings for other groups of professionals [200]. In general, studies found explicit racial bias to be less common than implicit racial bias [192].

### Effects of racism in healthcare on various treatment choices

This category includes 14 articles. All of these articles are from conducted in the USA and are quantitative except one mixed method article [95]. These articles examine the effect of racism in a variety of treatment choices. These treatment choices include various parts of healthcare including maternal and reproductive healthcare [203, 204], pediatric care [205], cardiovascular diseases [12, 95], end of life care [11], medical decisions in the emergency unit [61], oncology care [206] and immunotherapy [207]. In addition, other articles examine pain management [13, 208–210], HIV prophylaxis [211], atrial fibrillation treatment and dialysis treatment [212] and admission rate in an emergency unit [196].

#### Reports on the effects of racism on treatment choices

According to a quantitative study from the USA, Black women are more likely than White women to receive general anesthesia for cesarean delivery and to receive no analgesia for vaginal delivery [204]. Another study from the USA also shows that Black and Latinx healthcare users have lower rates of admission to the cardiology service compared to White patients [12]. Moreover, a study from the USA looking at stroke prevalence and treatment among various ethnicities found that Hispanics and Blacks have the highest risk of stroke in the population but are less likely to be prescribed anticoagulant medications than Whites [212]. In regards to end of life care, a study from the USA shows that Black healthcare users receive more burdensome end of life care in comparison to White healthcare users [11].

Another study from the USA that found implicit racial bias towards Whites in the emergency unit also found that implicit racial bias is associated with less serious diagnosis in regards to Black and Hispanic healthcare users when the emergency unit is crowded [61]. Another study from the USA looking at oncologists found that oncologists who measure high in implicit racial bias have shorter interactions with Black patients [206].

Moreover, a study from the USA also shows that White male physicians prescribe less pain medications to Black healthcare users compared to White users [208] and that healthcare professionals perceive Blacks to be biologically different than Whites and thus having differential reactions to pain [210]. Other studies from the USA also report differential pain management for Black healthcare users [13, 209] as well as less likelihood for prescribing HIV prophylaxis treatment for Black healthcare users [211]. Moreover, another study from the USA shows that African American are less likely to receive treatment with immunotherapy compounds independent of their insurance status [207]. A study from the USA shows that Newborn–physician racial concordance is associated with a significant improvement in mortality for Black infants and that these positive effects of physician newborn racial concordance manifest strongly in more complicated cases and when hospitals deliver more Black newborn [203]. Not all studies report differential treatment. For instance one studye from the USA found no racial differences in the admission rate in the emergency unit between various ethnic groups [196].

### Healthcare staff’s reflections on racism in healthcare

There are 15 articles that examine healthcare staff’s reflections on racism in healthcare [98, 155, 172, 213–224]. All of these articles are qualitative and are conducted in a variety of national contexts including the USA [214, 219, 222], Canada [217, 221, 224], Australia [216, 218, 220], UK [213], Sweden [155, 215], South Africa [223] and Israel [172].

Most of the articles examine various categories of healthcare staff. Articles examining specific categories of healthcare staff include articles examining nurses [172, 213, 215, 218] and care workers [155]. These articles explore general perceptions of racism in healthcare and do not focus on a specific ethnicity regarding the healthcare staff that are included.

#### Reports on healthcare staff’s reflections on racism in healthcare

In the UK [213], Canada [217], and the USA [219] studies show that healthcare staff tend to construct themselves as neutral and impartial and have difficulty in accepting that prejudice is part of healthcare interactions [213]. Similarly a study of nurses and physicians in Israel reports that the interviewed healthcare staff generally frame medicine as rational, neutral and based on objectivity [172] and that racism is seen as a matter of individual experiences rather than structural. In Sweden, a discrepancy between racialized minority and majority care givers is reported [155], regarding their views on racism in healthcare. While racialized minority care givers reflected on racism in healthcare, majority healthcare care givers tended to dismiss racism as existing in healthcare interactions. A Canadian study shows that healthcare staff who spent time working with Indigenous people show a more nuanced understanding of Indigenous people’s realities [221].

Studies from Australia report conflicting perceptions on racism in healthcare. While some studies show that healthcare staff are aware of racism as part of the Australian healthcare system specifically towards aboriginal healthcare users [69, 220], another study shows that healthcare staff are unable to define racism and that they often conflate racism with gender discrimination [218]. A study from the USA demonstrates that healthcare staff thought of ‘race’ as biological rather than a social construct [214].

### Antiracism training in healthcare

This review included 15 studies that evaluated various antiracist training programs and workshops for healthcare staff including students [225–239]. All articles are from research conducted in the USA except one article from the UK [234] and 2 from Australia [231, 239].

Most of these articles are quantitative (*n* = 7) [225–227, 230, 233, 235, 237]. Others used either qualitative methods (*n* = 5) [229, 232, 234, 236, 238] or were mixed methods (*n* = 3) [228, 231, 239].

Most of the antiracist training was conducted among medical students [225–227, 233–235, 239]. Other training was conducted in medical settings among faculty members [230, 231, 237, 238], among nurses [232], among physicians [236], and among various groups of healthcare staff [228, 229].

#### Reports on training on antiracism in healthcare

Difficulties in discussing racism in healthcare are reported in most of the studies. A qualitative study from the USA shows that medical faculty members do not discuss racism in healthcare in their workplace [238] and are uncomfortable about discussing racism. Another qualitative study from the USA illustrates that nurses Whiteness constituted a barrier in regards to teaching about racism in healthcare [232]. Another qualitative study from the USA also illustrates this difficulty and shows that healthcare staff found that discussions around racism as a structural and persistence issue was polarizing [229]. Similarly, a qualitative study from the UK demonstrates that there was generally an inadequate awareness of the meaning of multicultural care among medical students [234]. A quantitative study among various medical students from the USA [235] shows that students who graduated from an interdisciplinary pre-health curriculum identified relationships between structural factors and health outcomes more than pre-medicine science majors did.

Quantitative studies from the USA evaluating antiracist training show better understanding of racism in healthcare after the intervention [235], increased confidence and comfort in discussing and addressing racism [225, 227, 228, 233] and greater interest in receiving more antiracist training (Bi et al., 2020; Perdomo Joanna et al., 2019) [226, 237]. Healthcare staff who participate in antiracist interventions exhibit greater empathy towards racialized minorities according to a study in the USA [230] and one from Australia [239]. However, the study from Australia shows that while medical students demonstrate somewhat changed perceptions towards Aboriginal people and are more likely to challenge stereotypes, issues around racism were not resolved [239]. Moreover, the study from the USA [230] did not illustrate any change in healthcare staff’s implicit racial bias post-intervention as compare to pre-intervention.

## Discussion

This scoping review only includes English-language empirical articles examining racism in healthcare globally. To our knowledge, this is the first scoping review that reviews both qualitative and quantitative studies that explore racism in healthcare in various countries. The coding of the included articles showed that articles examine healthcare users’ and healthcare staff’s experiences of racism in healthcare, healthcare racial attitudes, and beliefs, effects of racism in healthcare on various treatment choices, healthcare staff’s reflections on racism in healthcare as well as articles examining antiracism training in healthcare.

Healthcare and national contexts notwithstanding, various racialized minorities seem to share commonalities in the way they experience racism as per the reviewed articles, especially regarding the more covert type of racism. Racialized minorities perceive their experiences of racism as consisting of inadequate care and a dismissal of their symptoms and suffering, a lack of respect, and a lack of power to negotiate in healthcare interactions [7, 36, 240, 241]. These experiences of racism result in a loss of trust in healthcare, higher unmet needs [242], and subsequently delaying seeking healthcare [243]. Moreover, studies report that healthcare staff tend to homogenize racialized minority healthcare users, to view them as irrational, difficult and frustrating, and as too emotional and dramatic [14, 181, 182]. Studies looking at implicit racial bias show that healthcare staff may exhibit racial bias in favor of majority groups [191, 244], such that minorities receive deprioritized diagnoses and treatment that [61]. Studies that look at differential treatment between racialized minorities and majority groups also illustrate inadequate care regarding various treatment choices such as pain medications, HIV prophylaxis [211], cardiovascular disease [95], perinatal care [203], end of life care [11], and other indicators [209, 245]. Moreover, racialized minority healthcare staff also experience racism both from healthcare users and other healthcare staff [149, 158]. There is an expectation that racism from healthcare should be tolerated, resulting in emotional depletion and mental health issues, as healthcare staff often do not find space to discuss their experiences of racism in their workplace [153].

While racism persists in healthcare and operates in various dimensions, healthcare staff nonetheless construct themselves and the healthcare they provide as objective and neutral [172, 213, 217, 219]. Studies show that healthcare staff find it difficult to discuss racism and accept racism as part of healthcare interactions. While some studies show some healthcare staff awareness of racism as a structural issue in healthcare [221], confusion regarding how to define racism and ‘race’ is also apparent [69, 220]. This is also shown by studies that evaluate antiracist training. Healthcare staff participating in such training often state that they do not discuss racism in their workplace and find it uncomfortable to discuss racism [232, 238]. Although antiracism training shows that healthcare staff exhibit greater empathy towards racialized minorities, it is unclear whether such educational efforts effectively alter racial bias and attitudes.

### Knowledge gaps in the reviewed articles

#### Lack of articles on racism in healthcare in European contexts

The articles reviewed here are dominated by research from the USA, which constitutes 67% of the articles scoped, followed by UK and Canada (7%). The domination of data from the USA may be explained by the availability of racial category data in the USA. In the USA, ‘race’ is a legal category, enabling data to be readily available for research, which is not the case in many European countries. The small number of European studies, mainly from the UK, shows that racism in healthcare is also a problem in these countries. However, with the exception of the UK, it is presently difficult to offer any conclusive evidence of racism in healthcare in Europe. This lack of evidence regarding racism exists despite evidence of racial/ethnic inequalities between foreign-born healthcare users and natives in Europe [246] and reports of discontent and othering processes in healthcare encounters. This could be due to the sensitive and politicized nature of racism and the construction of Europe as exceptional and antiracist [28, 247]. With the rejection of ‘race’ as a scientific category, following the Second World War, ‘race’ and racism have been largely occluded from European social formations, political and public discourse [247], while integration of migrants has been the main policy focus [248]. This focus on integration, with racism constructed as an issue of the past, makes discussions around racism in Europe difficult [249]. This difficulty informs research on racism in healthcare and is reflected in the seemingly sparse research in Europe on the issue in contrast to the USA, where discussions around racism are more common. In addition to this, many of the articles that reported on racism in healthcare from European settings did not set out to examine racism in healthcare. For instance, research setting out to examine barriers to and facilitators for racialized minorities seeking in healthcare in European settings, ended up reporting racism since racialized minorities discussed experiencing racism. This reflects that racism is experienced but may be under researched due to the difficulties of discussing racism in a European setting. Hence, there is a need to do more research regarding racism in healthcare in European settings both from the perspectives of healthcare users and healthcare staff.

#### Research on racism in healthcare is descriptive, ahistorical and atheoretical

The research on racism in healthcare is fragmented and scattered across disciplines and healthcare contexts. Thirty-seven percent of the included articles did not set out to examine racism in healthcare and hence framed their research in terms other than racism. Nevertheless, the majority of the remaining 67% of the articles that had racism as their main focus, except a few articles [14, 32, 35, 36, 43, 46, 48, 50, 51, 53, 69, 76, 91, 96, 98, 102, 118, 123, 127, 142, 148, 149, 152, 156, 160, 162, 165, 169, 172, 175, 189, 192, 218, 221–224, 241, 250–256] lacked a conceptualization of racism and ‘race’ and did not include a definition of these concepts. As research lacks a way to define and conceptualize racism in healthcare, the majority of the studies treat ‘race’ and racial categories as real, ahistorical, and pre-constituted categories. There is a general lack of attention to and illustration of the historical context within which these racial categories have been formulated. Put in other words, research tends to study ‘race’ instead of looking at racialization processes and the resulting racism that subsequently constructs an idea of ‘race’ (Nazroo et al., 2020). Furthermore, studies, especially quantitative studies from the USA looking at differential treatment across racial categories, tend to conflate the terms ‘race’ and ethnicity. For instance, Black and White are considered racial categories in the USA, while Hispanic is considered an ethnic category [257]. In the reviewed articles, these categories are often examined together. Since these categories are, for the most part, not defined, it is unclear why they were chosen, what they mean and what the results that show differential treatment along these categories convey in regards to racialization processes in healthcare. Robert Miles [30] argues that racism is a historical process of domination where racialization is a central part of modern societies. Omi and Winant [258] define racialization as ‘the sociohistorical process by which racial categories are created, inhabited, transformed, and destroyed (…) Race is a matter of both social structure and cultural representation’ (p: 55–56). As such, racism operates in a wider system of racialization in which racial categories are produced and given meaning within racial hierarchies [259]. Robert Miles suggests that a way to understand and conceptualize racism is to view it as a process of racialization rather than invoking ‘race’ since ‘race’ is by default a vacuous ontological category that is produced by racism [30]. When ‘race’ is invoked in a non-critical and ahistorical manner, there is a risk of producing racial categories as fixed and unchanging biological classifications instead of the unstable social constructs that they are. Moreover, without a historical understanding of the meaning-making of racial categories, it becomes problematic since there is a risk that racialization is emphasized and reproduced in research.

Ahistorical and atheoretical approaches to research entails that many of the studies included in this scoping review frame their research around the specific illnesses/diseases or symptoms that they study rather than around racism. For instance, studies that look at HIV prophylaxis, e.g. [211] or opioid prescription [260] would frame their study around these specific illnesses rather than racism. While this is a valid way of framing studies, the consequence of this in regards to the general nature of the research is that the object of research becomes the illness or condition studied rather than racism itself which remains untheorized. While it is important to document inequalities in treatment and health indicators, it is well established that racism is an integral part of modern institutions and structures [1]. Research around racism in healthcare needs to move beyond describing the existence of racism to theoretically explaining and conceptualizing how racism actually works in healthcare and the racialization processes that lead to material inequalities in healthcare. In order to do this, research on racism in healthcare should engage with sociological research regarding the meaning-making of racial categories and racialization processes.

This review confirmed that the dominant professional discourse in healthcare portrays healthcare staff as ‘natural history scientists’ who are not affected by bias, as reflected by the discrepancy between healthcare staff’s image of themselves as objective on the one hand [172, 213, 217, 219], despite evidence of of racial bias [185, 187, 261] as well as healthcare users’ experience of racism in healthcare settings [32, 38] on the other hand. For the most part, this discrepancy is left underexplored as the main focus of articles is to document experiences of racism. Sociological research on racial denial (van Dijk, 1992; van Dijk, 1999) [262, 263], colorblind racism, and racism without racists [4] may aid in explaining why racism remains a difficult issue to discuss, even in USA where a majority of studies of this topic are conducted. Sociological research on contemporary racism may also illuminate why some articles show that healthcare staff tend  to deny the existence of racism in healthcare even with evidence of racial inequalities. ‘Racism without racists’ understanding of contemporary racism [4] offers a framework that explains how racism persists in healthcare interactions even when healthcare staff constructs themselves and healthcare as neutral. Hence, sociological research on racism and racialization may help research on racism in healthcare to examine how racial inequality can be reproduced through supposedly non-racial practices such that colorblind racism can become normalized in healthcare.

#### Research on antiracism in healthcare and future implications

There has been a huge increase in the volume of research on racism in healthcare from 2017 onwards, amounting to more than 100 articles that were found through this review. This is positive as it reflects an academic increase in the topic of racism in healthcare. However, this increase in volume does not imply an improved quality since these articles report on the same dimensions of racism that have already been reported on in previous years, even when they report on different healthcare contexts. The research remains descriptive, ahistorical and lacking definitions of key concepts. However, an exception to this is an increasing, although still limited, amount of research on antiracism interventions in healthcare. This research is an emerging dimension essential in dealing with racism in healthcare and needs to be further developed. However, this research remains atheoretical as well. If racism is often produced through unconscious processes as evidenced by research showing the existence of implicit racial bias of healthcare staff, how will antiracist interventions speak to the unconscious nature of racism? Some of the studies examining antiracist training show that although healthcare staff’s empathy towards racialized minorities increases after these interventions and that healthcare staff feel more comfortable discussing racism, racism remains a polarizing issue [239, 264].

Additionally, one study shows that implicit racial bias pre-and post-intervention remains significantly unchanged [230], which is to be expected if racism is understood as an outcome of historical processes and deeply embedded in social institutions. The question remains as to how to design and implement effective antiracist interventions in healthcare. Nazroo [6] argues that ‘any consideration of racism must necessarily focus on the structural, macro, level (…) Here, the legacies of historical regimes of colonialism, race-based slavery and apartheid interact with current processes of globalisation, migration and governance, continuing to shape present day inequalities in accessing key economic, physical and social resources’ (p: 265). As such, antiracism training and intervention must find a way to solicit reflections of these historical regimes over a longer period. One way of doing this is to implement educational strategies within medical, educational and organizational settings to make visible structures of racial domination and to have continuous conversations and dialogues on the issue of racism and its intersections with other systems of domination.

### Limitation of this review

Although the review followed a systematic method, it is possible that not all articles were incorporated. Additionally, as articles were only included if the search terms were used in the abstract or title, articles which discussed racism in the text without naming it in the title or abstract may have been excluded. Furthermore, articles discussing racism that used terms other than racism or discrimination such as bias, prejudice and stereotypes were also not included in this review. No screening of references in the included articles was conducted. The results of this review are based on English-language empirical research reports, which excludes articles in languages other than English that may discuss racism. This partially explain why the majority of the articles are from English speaking countries.

## Conclusion and key recommendations

**Fig. 5:**
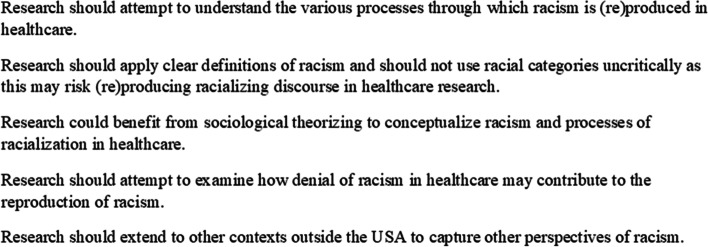
Key recommendations for future research on racism in healthcare

Research on racism in healthcare show that racism operates in various dimensions between healthcare staff and users and affects treatment and diagnosis in various health indicators. In Fig. 5 above, key recommendations on future research on racism based on the results of the review, are provided. There has been an increase in the volume research from 2017 onwards. However, research is mainly descriptive, atheoretical, and uses racial categories critically, as if they were fixed and ahistorical. Hence, research tends to ignore racialization processes in healthcare and makes it difficult to conceptualize racism in healthcare and understand how racism is produced in healthcare. Research on racism in healthcare could benefit from sociological research on racism and racialization to explain how overt racism is produced and how racism is normalized and hidden behind supposedly non-racial practices in healthcare. Additionally, research is dominated by the USA, and it is imperative that research is also conducted in other geopolitical contexts.

## Supplementary Information

**Additional file 1.** Prisma checklist. Preferred Reporting Items for Systematic reviews andMeta-Analyses extension for Scoping Reviews (PRISMA-ScR) Checklist.

**Additional file 2:** **Table 1.** Summary of articles describing racismin healthcare included in scoping review: title, authors, year, location, aim,methods and findings.

## Data Availability

All articles included in this review are available in a table included as an additional file.
