# Supplementary material for: Racism in healthcare: a scoping review
Source: BMC Public Health. 2022 May 16;22:988. doi: 10.1186/s12889-022-13122-y (PMC9112453; doi:10.1186/s12889-022-13122-y)
Supplement: Supplementary file 2 — Additional file 2: Table 1. Summary of articles describing racismin healthcare included in scoping review: title, authors, year, location, aim,methods and findings. [file 12889_2022_13122_MOESM2_ESM.docx]

Table S2: Summary of articles describing racism in healthcare included in scoping review: title, authors, year, location, aim, methods and key findings in regards to racism in healthcare.

Methods that are not clearly stated in the reviewed articles are not reported in the table and are stated as unclear.

| ***Qualitative articles examining healthcare users’ experiences of racism in healthcare (n=69)*** | | | | | |
| --- | --- | --- | --- | --- | --- |
| **Article title and author/s** | **Year of publication** | **Geographical location** | **Aim of article** | **Methods** | **Key findings in regards to racism in healthcare** |
| Perceptions and Experiences with Nursing Care: A Study of Pakistani (Urdu) Communities in the United Kingdom  Cortis, Joseph Domenic | 2000 | UK | To explore experiences with nursing care from the perspectives of a Pakistani community in the UK. | n=30 males and 25 females with Urdu-speaking Pakistani Muslim adults. | - Nurses were expected by healthcare users to be friendly and care was expected to be equitable. However, experiences of stereotypes held by nurses towards the Pakistani community existed and racism was seen as a cause of perceived lack of congruence between expectations and experiences with nursing care. Being Black was the reason for racism, as articulated by the participants. |
| First Nations women’s encounters with mainstream health care services  Browne, A. J., & Fiske, J. A. | 2001 | Canada | Exploring the experiences of First Nation women with healthcare. | n=10 interviews with First Nation women. | -Participants reported that their symptoms were dismissed or trivialised and attributed this to their ethnicity. This was compounded with what these women were taught in Catholic school to not complain and express pain.  -In general, participants experienced racism in healthcare as part of a larger structure of racism given the colonial context in Canada. --Participants were also treated as less deserving to be mothers compared to other groups and experienced racism as marginalisation and as being intruding.  -Participants adopted strategies such as transforming oneself to gain credibility when navigating healthcare. |
| Faith and feminism: how African American women from a storefront church resist oppression in healthcare  Mary Abrums | 2004 | USA | How African American women resist racism in healthcare? | Ethnographic study in a small church. | -A Black feminist standpoint epistemology was used to analyse women’s resistance to racism. Religious beliefs were used to help women cope and resist racism.  -To navigate healthcare women employed strategies included dressing well, taking the initiatives and asking questions, working hard, praying and having a high self-esteem. |
| The last mile of the way: understanding caregiving in African American families at the end-of-life.  Turner, W. L., Wallace, B. R., Anderson, J. R., & Bird, C. | 2004 | USA | To explore African American’s perceptions on end of life caring homes. | n=88 African American family caregivers. | -Participants reported distrust to formal care due to years of exclusion, racism and discrimination.  -Faith was perceived as important for dealing and coping with stress. |
| African American women’s coping with health care prejudice  Benkert, R., & Peters, R. M. | 2005 | USA | To examine African American women’s experiences with racism in healthcare and their coping mechanisms. | n=20 African American women. | -Participants were mostly of lower socioeconomic status and lived in segregated areas with minimum contact with White people.  -Both overt and covert racism was reported by participants. Participants also reported being scolded by healthcare providers and perceived to receive poor treatment due to racism.  -Various coping mechanisms were developed such as getting angry, learning to  unlearn (involved turning to God, giving the provider the benefit of the doubt), being assertive, and walking away. |
| Racial differences in attitudes regarding cardiovascular disease prevention and treatment: a qualitative study  Woodard, L. D., Hernandez, M. T., Lees, E., & Petersen, L. A. | 2005 | USA | To explore coronary heart disease healthcare experiences and beliefs of African-American and White patients to elicit potential causes of racial disparities in coronary heart disease outcomes. | n= 24 patients (10 African Americans and 14 Whites). | -Both groups expressed similar knowledge in regards to coronary heart diseases and stated the importance of having a good physician-patient relationship.  -African Americans also stated that they experienced racism in healthcare, which was seen as stressful. |
| Understanding African Americans’ Views of the Trustworthiness of Physicians  Jacobs, E. A., Rolle, I., Ferrans, C. E., Whitaker, E. E., & Warnecke, R. B. | 2006 | USA | To explore African American’s views on trust to healthcare physicians. | n=66 African American adults (34 men and 32 women). | -Participants reported interpersonal and technical competence of healthcare providers as important. Lack of these types of competence influenced trust to healthcare providers.  -Participants often described how physicians focused on profit rather than their care. They also reported that race influenced trust in physicians.  -Distrust inhibited care-seeking, and resulted in a change in physician and at times led to noncompliance. |
| With or without intent: how racial disparities prevent effective implementation of care  Subban, J. E., Terwoord, N. A., & Schuster, R. J. | 2008 | USA | To explore how racial barriers limit the effective implementation of health care by examining the barriers that affect health care among African-Americans in Dayton, Ohio. | n=18 healthcare users (16 African Americans and 2 Whites) with low socioeconomic status. | -Participants reported that socioeconomic status (unemployment, limited earning and lack of insurance) were barriers to accessing healthcare.  -Although participants commented on racism, these comments on racism were not always directly related to healthcare services and were more about the historical struggle of blacks in the USA. |
| What do non-English-speaking patients value in acute care? Cultural competency from the patient’s perspective: a qualitative study  Garrett, P. W., Dickson, H. G., Whelan, A. K., & Roberto-Forero | 2008 | Australia | To locate conceptions of cultural competence within the experiential domain of the non-English speaking patient. | n=59 hospital patients and carers of patients with limited English. | -The majority of participants were positive about their healthcare experience. However, powerlessness was identified as central to many of their experiences.  -Language facilitation was the most common concern as well being attentive to cultural norms.  -In this study racism was only mentioned by Muslim Arabic speaking groups and was specifically connected to wearing Hijab. |
| “We’ve fallen into the cracks”: Aboriginal women’s experiences with breast cancer through photovoice  Poudrier, J., & Mac-Lean, R. T | 2009 | Canada | To explore Aboriginal women who completed cancer treatment experience with healthcare | Photovoice was used with 12 Aboriginal women’s who completed cancer treatment. | -Participants reported the importance of Aboriginal identity and traditional spiritual beliefs in their experience with breast cancer.  -Participants also reported experiencing racism in healthcare, although positive experiences were also identified. Racism was articulated through the use of racial slurs by healthcare providers, and patronising attitude. These experiences contributed to the invisibility of women in healthcare encounters. |
| Needs and Preferences for Receiving Mental Health Information in an African American Focus Group Sample  Mishra, S. I., Lucksted, A., Gioia, D., Barnet, B., & Baquet, C. R. | 2009 | USA | To examine African Americans experiences with mental health information. | n=42 interviews with African Americans. | -Participants expressed the need to receive adequate information in regards to mental illnesses in a way that involves ensuring anonymity, confidentiality and support.  -In regards to barriers to healthcare, issues related to stigma due to mental illnesses as well as perceptions of racism were reported by participants. Perceptions of racism were related to historical racism against African Americans as well as recurrent racism and mistrust of the healthcare system. |
| Perceived racial discrimination in clinical encounters among African American hypertensive patients  Greer, T. M | 2010 | USA | To look at the experiences of racism in healthcare among African American people with hypertension. | n=6 focus group discussions including 47 African American patients. | - Results show that participants perceived good providers as those who could communicate effectively, were responsive to immediate complaints and who ensured equity in care.  -Experiences of racism were perceived as avoidance of touch by healthcare providers, assumptions about African American’s ability to afford services as well as apathy in reaching diagnosis.  -There was a general mistrust of White healthcare providers and the healthcare system which was seen as unfair to  African Americans. |
| Race and shared decision-making: perspectives of African-Americans with diabetes  Peek, M. E., Odoms-Young, A., Quinn, M. T., Gorawara-Bhat, R., Wilson, S. C., & Chin, M. H | 2010 | USA | To explore the reasons that may lie behind racial disparities in diabetes among African Americans. | N=44 individual interviews with African Americans | -The majority of participants stated that race did not influence the patient/provider communication or shared decision making. –  -Most participants framed their stories in terms of what should occur in an ideal relationship instead of experiences of racism.  -None of the participants reported any experiences of racism within healthcare encounters. However, participants did describe hypothetically how race may influence shared decision making in healthcare.  -Participants discussed how they perceived African Americans as less forthcoming in regards to their symptoms, less likely to speak up to authority and less likely to adhere to treatment. |
| Strategic approaches to enhanced health service delivery for Aboriginal and Torres Strait Islander people with chronic illness: a qualitative study  Aspin, C., Brown, N., Jowsey, T., Yen, L., & Leeder, S | 2012 | Australia | To identify barriers and facilitators to care and support for Aboriginal and Torres Strait Islander people with chronic illness. | n=19 in-depth interviews with Aboriginal and Torres Strait Islander participants who had chronic illness and/or cared for a family member with chronic illness. | -Participants encountered both positive and negative encounters in healthcare.  -Participants encountered racism in healthcare but also discussed racism in other welfare institutions including education and social services. These experiences of racism were linked with feelings of frustration, sorrow and dismay and a feeling of being seen as small. These feelings made it hard for patients to focus and adhere to treatment. In addition to that. participants reported being judged by staff and being offended. This influenced their communication with healthcare providers.  -Participants reported strategies to cope with racism including recruiting aboriginal healthcare providers, having family and peer support and having long-term relations with healthcare providers. |
| Using Illness Narratives to Explore African American Perspectives of Racial Discrimination in Health Care  Ross, P. T., Lypson, M. L., & Kumagai, A. K. | 2012 | USA | To explore African Americans experiences with healthcare in connection to racism. | n=12 African American adults (4 men and 8 women). | -Participants described that they received not only differential treatment but also inferior treatment in comparison to other individuals.  -Participants perceived that White patients are listened to more, addressed more formally and that healthcare professionals assumed the worst about them (assuming they were poor, lacked health insurance and health knowledge and faked symptoms).  -Blatant racism was reported as well as nonverbal behaviour such as rolling the eye was also identified as racism.  -Participants developed mechanisms to cope with racism in healthcare including being assertive, employing respectability tactics such as making sure providers knew their social status, educational background, or professional occupation as well as being informed about their illness and preparing list of questions to show healthcare providers that they were knowledgeable. |
| Help bring back the celebration of life: a community-based participatory study of rural Aboriginal women’s maternity experiences and outcomes.  Varcoe, C., Brown, H., Calam, B., Harvey, T., & Tallio, M. | 2013 | Canada | To explore the experiences of Aboriginal women in rural areas with maternity care. | n= Over 100 Aboriginal women | -Participants reported having challenging experiences within healthcare encounters, connected to maternity care, which included, diminishing local maternity care choices and economic circumstances and racism.  -Racism was viewed as the constant companion of colonialism and was reported as being overt articulated through derogatory remarks against Aboriginal women and their babies.  -Racism was also experienced during the birthing experience where healthcare providers were perceived as dismissive.  -Racism resulted in a stressful relations with healthcare providers. |
| What is patient-centered care really? Voices of Hispanic prenatal patients  Bergman, A. A., & Connaughton, S. L | 2013 | USA | Perception of Hispanic prenatal patients on patient centred care. | n=48 Hispanic prenatal patients. | -The experience of a friendly relationship with providers was the most commonly discussed theme when discussing patient centred care.  -Providers were expected to be thorough and provide effective treatment.  -The availability of providers who speak Spanish and providers who could give clear information was also stressed.  -Elimination of racism was also perceived as an important part of patient centred care. |
| “You learn to go last”: perceptions of prenatal care experiences among African-American women with limited incomes  Salm Ward, T. C., Mazul, M., Ngui, E. M., Bridgewater, F. D., & Harley, A. E | 2013 | USA | -To explore the views of African American women with low incomes’ experience with prenatal care. | n=29 African American women. | -Participants reported three areas of perceived discrimination namely, discrimination based on insurance or income status, discrimination based on race, and lifetime experiences of racism.  -Participants discussed receiving differential treatment due to income and type of insurance which they stated may have influenced quality of care.  -Participants also reported racism in healthcare encounters. Racism was both direct and subtle such as negative assumptions by healthcare providers. |
| Facilitators and barriers to help-seeking for breast and cervical cancer symptoms: a qualitative study with an ethnically diverse sample in London  Marlow, L. A. V., McGregor, L. M., Nazroo, J. Y., & Wardle, J. | 2014 | UK | To explore experiences of seeking care for breast and cervical cancer symptoms among a diverse sample in London | n=54 healthy women from various ethnic minorities. | -Two women (one Black Caribbean and one Pakistani woman) reported experiences of perceived racism as a barrier to seeking healthcare. |
| Discordant indigenous and provider frames explain challenges in improving access to arthritis care: a qualitative study using constructivist grounded theory  Thurston, W. E., Coupal, S., Jones, C. A., Crowshoe, L. F., Marshall, D. A., Homik, J., & Barnabe, C | 2014 | Canada | To explore the experiences of indigenous people with arthritis when seeking healthcare | n= 16 patients.  n=15 healthcare providers. | -Participants talked about having to “toughing it out” and that racism shaped their healthcare encounters and experiences. |
| Exploring the Cervical Cancer Screening Experiences of Black Lesbian, Bisxeual, and Queer Women: The Role of Patient-Provider Communication  Madina Agénor, Zinzi Bailey, Nancy Krieger, S. Bryn Austin & Barbara R. Gottlieb | 2015 | USA | To explore LBQ blacks experiences with cervical cancer screening | n=18 Black LBQ women | -Participants emphasised the importance of trust in the healthcare meetings.  -Heteronormative healthcare providers’ perceptions were perceived as problematic and were related to patients’ sexual orientation as well as their race and class. –  -Fear was also experienced due to multiple forms of discrimination. |
| Visibility and Voice: Aboriginal People Experience Culturally Safe and Unsafe Health Care.  Hole, R. D., Evans, M., Berg, L. D., Bottorff, J. L., Dingwall, C., Ale1is, C., … Smith, M. L. | 2015 | Canada | To explore aboriginal people’s experiences with healthcare. | n=28 Aboriginal community members (5 men and 23 women). | -Participants discussed positive experiences in healthcare encounters in regards to being visible which for them meant being heard and respected as Aboriginals and as human.  -In regards to the negative experiences with healthcare, Aboriginal people in the study highlighted the historical dimension within which healthcare is situated i.e., healthcare’s colonial perspectives.  -Participants experienced being ignored and dismissed due to them being Native. This made the feel invisible. |
| Mental Illness Discrimination in Mental Health Treatment Programs: Intersections of Race, Ethnicity, and Sexual Orientation  Holley, L. C., Tavassoli, K. Y., & Stromwall, L. K. | 2016 | USA | To examine experiences with mental healthcare among patients with mental health illnesses. | n=20 people with mental illness. | -Participants discussed racism, heterosexism, and mental illness discrimination. Although discrimination was encountered due to sexual orientation, non-White participants described also experiencing racism in healthcare encounters. These participants talked about receiving differential treatment in comparison to White patients. |
| They treated me like crap and I know it was because I was Native”: The healthcare experiences of Aboriginal peoples living in Vancouver’s inner city  Goodman, A., Fleming, K., Markwick, N., Morrison, T., Lagimodiere, L., Kerr, T., & Western Aboriginal Harm Reduction Society | 2017 | Canada | To explore healthcare experiences of Aboriginal people who use illicit drugs or illicit alcohol. | n=30 interviews were conducted with Aboriginal people living in segregated and poor neighbourhoods. | -Participants reported being treated badly and differently because they were native.  -Participants reported being treated with suspicion and perceived that healthcare providers were more concerned with affirming their assumptions about illicit drug use than providing medical care.  -Feeling of discrimination led patients to either put up with discrimination or to avoid seeking healthcare as well as not to disclose correct information on drug use. |
| Facilitators and Barriers to Dental Care Among Mexican Migrant Women and Their Families in North San Diego County  Velez, D., Palomo-Zerfas, A., Nunez-Alvarez, A., Ayala, G. 1., & Finlayson, T. L | 2017 | USA | To look at barriers to accessing dental care as perceived by Mexican migrants. | n=6 Focus group discussion with 53 Mexican migrants. | -Immigration status was the main barrier to accessing dental care followed by disrespect by staff. In addition to that, high cost, lack of insurance and long waiting times were also mentioned as barriers by participants.  -Discrimination due to race and class were also mentioned as barriers to healthcare. |
| US healthcare experiences of Hispanic patients with diabetes and family members  Amirehsani KA1, Hu J1, Wallace DC1, Silva ZA1, Dick S1, West-Livingston LN1, Hussami CR1. | 2017 | USA | To explore the experiences of Hispanic patients with diabetes with healthcare. | n=172 Hispanic adults. | -Participants had concerns with receiving adequate information, receiving attentive care where providers listen to their needs as well as wanting better continuous care preferably with the same provider.  -Participants also reported perceived discrimination which was viewed as institutional in regards to lack of insurance but also reported racism from healthcare providers. |
| Critical race theory as a tool for understanding poor engagement along the HIV care continuum among African American/Black and Hispanic persons living with HIV in the United States: a qualitative exploration  Robert Freeman, Marya Viorst Gwadz, Elizabeth Silverman, Alexandra Kutnick, Noelle R. Leonard, Amanda S. Ritchie, Jennifer Reed and Belkis Y. Martinez | 2017 | USA | To explore the experiences of African American/Black and Hispanic persons living with HIV using critical race theory. | n=37 participants (African American and Hispanic patients with HIV) were recruited as part of a larger project. | -Racism was seen as the primary reason for having a subordinate position in healthcare although poverty and unequal resources also informed participants’ experiences with healthcare.  -Participants experienced a distrust of medical institutions and healthcare providers as well as being excluded from health decision making process.  -The way participants were treated due to their HIV illness was considered to be dehumanising.  -Racism was described as both institutional regarding lack of resources as well as part of a dehumanisation processes.  -Racism resulted in a direct distrust of healthcare providers. |
| Performing Black womanhood: a qualitative study of stereotypes and the healthcare encounter  Sacks, T. K | 2018 | USA | To look at how African American women cope or deal with racism in healthcare. | n=19 middle class African American women. | -Participants deal with racism through dressing the part and through talking intelligently to establish a good relation with healthcare providers. Conducting research on their illnesses to appear knowledgeable prior to the healthcare encounter was also a strategy used. |
| Health Care of experiences of pregnant, birthing and postnatal women of color at risk for preterm birth  McLemore MR, Altman MR, Cooper N, Williams S, Rand L, Franck L. | 2018 | USA | To explore pregnant related healthcare experiences of women of color. | n=54 women of color were interviewed. | -Participants reported feelings of disrespect and feeling dismissed and treated rudely because of their race but also because of their marital status, and class.  -Participants were not surprised that they experienced racism as they viewed racism as a common experience throughout their daily lives.  -Participants also reported stress interactions with healthcare providers as well as unmet needs, limited support and uncoordinated services. |
| Obstetric Racism: The Racial Politics of Pregnancy, Labor, and Birthing  Dána-Ain Davis | 2019 | USA | To analyse birth stories of Black women living in the USA. | n =3  Three cases of birth stories by African American women. | **-**Participants reported being dismissed and not taken seriously during their pregnancy and birth.  -Participants’ stories were framed in the article as obstetric racism. |
| Sub-Saharan African immigrant women's experiences of (lack of) access to appropriate  healthcare in the public health system in the Basque Country, Spain  Pérez-Urdiales I, Goicolea I, Sebastián MS, Irazusta A, Linander | 2019 | Spain | To explore Sub-Saharan immigrant women’s perceptions and experiences of healthcare. | n =14 women from 8 Sub-Saharan African countries. | -Participants reported fear of the health system, which was perceived as unfriendly towards immigrants.  -Participants also discussed mistreatment in healthcare due to racism where being, immigrant, and Black may influence healthcare interactions. |
| Shared decision-  making around anal cancer screening among black bisexual and gay men in the USA  Acree ME, McNulty M, Blocker O, Schneider J, Williams H'S | 2019 | USA | To evaluate the relationship between intersectionality and shared decision-making around anal cancer screening in Black gay and bisexual men. | n =45 participants. | - Some participants described how Black men were reluctant to engage and trust healthcare due to past medical experimentation on Black people as well as ongoing health inequalities.  - Some participants reported that Black people were more likely to experience provider racial bias and more likely to receive less adequate medical information in comparison to White gay men. |
| Access to primary healthcare for asylum seekers and refugees: a qualitative study of service user experiences in the UK.  Kang C, Tomkow L, Farrington R | 2019 | UK | To examine asylum seekers and refugees’ experiences accessing primary  healthcare in the UK in 2018. | n =18 interviews with asylum seekers and refugees. | -Some participants reported experiencing racism from healthcare providers. |
| Community Based Participatory Research (CBPR): A Dynamic Process of Health care, Provider Perceptions and American Indian Patients' Resilience  Elizabeth Hulen, Lisa J. Hardy, Nicolette Teufel- Shone, Priscilla R. Sanderson, Anna L. Schwartz, R. Cruz Begay | 2019 | USA | To explore American Indian resilience in navigating healthcare. | n =39 American Indians and 22 healthcare providers who serve American Indians. | -American Indians described racist remarks and negative stereotypes in their interactions with healthcare providers.  -Healthcare providers perceived American Indians as unable to understand health issues and as unmotivated. |
| “What I Wish My Doctor Knew about My Life’: Using Photovoice with Immigrant Latino Adolescents to Explore Barriers to Healthcare.”  Lightfoot, Alexandra F., Kari Thatcher, Florence M. Simán, Eugenia Eng, Yesenia Merino, Tainayah Thomas, Tamera Coyne-Beasley, and Mimi V. Chapman | 2019 | USA | To explore immigrants Latino Adolescents’ barriers to healthcare. | n =13 using photovoice methodology. | -Participants described their relationship with healthcare providers as a closed door where negative stereotypes about their racial groups were common.  -Participants also reported how healthcare providers pressured them to take pregnancy tests even when they reported not being sexually active. |
| “A gay man and a doctor are just like, a recipe for destruction”: How racism and homonegativity in healthcare settings influence PrEP uptake among young Black MSM  Quinn K, Dickson, Gomez J, Zarwell M, Pearson B, Lewis M. | 2019 | USA | To examine how Black MSM perceive experiencing healthcare and perceptions on PrEP uptake. | n = 44  Focus group discussions with Black MSM | -Participants reported being dismissed and deprioritised due to their racial category.  -Participants’ experiences with racism in healthcare and mistrust of the healthcare system made it difficult for the participants to discuss their sexual orientation with healthcare providers.  -Participants also described that racism influenced their trust in the healthcare system as a whole and their uptake of PrEP. |
| Healthcare Experiences of Transgender People of Colors  Howard SD, Lee KL, Nathan AG, Wenger HC, Chin MH, Cook SC | 2019 | USA | To investigate how the experiences of healthcare among transgender people of color are shaped by both race/ethnicity and gender identity. | n = 22 in depth interviews  n = 17 in 2 focus group discussions. | -Participants discussed how they felt that their experiences in healthcare were informed by their racial identities.  -Participants discussed negative assumptions by healthcare providers based on negative racial views held by providers.  -Participants also reported trying to seek providers who matched their racial identities and who were LGBTQ friendly. |
| "The fear of being Black plus the fear of being gay": The effects of intersectional stigma on PrEP use among young  Black gay, bisexual, and other men who have sex with men  Quinn K, Bowleg L, Dickson-Gomez J | 2019 | USA | To understand how the intersection of racism, homonegativity, HIV stigma, and Pre-exposure prophylaxis stigma collectively affect HIV risk and prevention opportunities for young Black gay men. | N=44 young Black gay men. | -Most of the participants discussed the intersection of their race and sexual identity and how they were mistreated for being black as well as being gay. |
| Understanding the role of past health care discrimination in help-seeking and shared decision-making for depression treatment preferences  Progovac, A. M., Cortés, D. E., Chambers, V., Delman, J., Delman, D., McCormick, D., . . . Cook, B. L | 2020 | USA | To understand the role that past health care discrimination plays in shaping help-seeking for  depression treatment and receiving preferred treatment modalities. | n =21 adults with depressive symptoms from various ethnicities. | -Participants of color reported past discrimination when accessing healthcare due to their ethnicity. These participants also reported discrimination due to ethnicity experienced by their friends and family members.  -Experiences of discrimination based on ethnicity were connected to feelings of frustration and at times fear and terror in healthcare interactions. |
| Indigenous mothers' experiences of using acute care health services for their infants  Wright AL, Jack SM, Ballantyne M, Gabel C, Bomberry R, Wahoush O | 2019 | Canada | To develop an understanding of how Indigenous mothers experience selecting and using health services for their infants. | n =19  Interviews and group discussions. | -Experiences of racism were reported and led to loss of trust in healthcare. |
| Information and power: Women of color's experiences interacting with health care providers in pregnancy and birth  Altman MR, Oseguera T, McLemore MR, Kantrowitz-Gordon I, Franck LS, Lyndon | 2019 | USA | To explore how interactions with healthcare providers were perceived by women of color in the context of pregnancy and birth care. | n =22 individual interviews with women of color. | -Participants perceived that healthcare providers consciously or unconsciously used their power as providers to limit information sharing in regards to pregnancy and birth issues.  -Participants reported differential healthcare due to racism, poverty, education and public insurance. |
| Breaking down the barriers: Understanding migrant workers’ access to healthcare in Malaysia  Loganathan T, Rui D, Ng CW, Pocock NS | 2019 | Malaysia | To explore barriers to healthcare access faced by documented and undocumented  migrant workers in Malaysia. | n =17 with informants from civil  society organizations, trade unions, academia, medical professionals, as well as migrant  workers and their representatives | -Discrimination in healthcare and from healthcare providers was documented among other barriers experienced by migrant workers in Malaysia.  -Participants reported that healthcare providers tend to be negligent towards migrant workers. |
| “Just because you have ears doesn’t mean you can hear” perception of racial-ethnic discrimination during childbirth  Janevic, T., Piverger, N., Afzal, O., & Howell, E. A. | 2020 | USA | To examine the impact of perceived racial-ethnic discrimination on patient-provider communication among Black and Latina women giving birth in a hospital setting. | n =27 Black and Latina women who gave birth in a hospital setting. | -Participants reported experiencing racism in healthcare interactions during childbirth and felt dismissed by nurses.  -Participants reported that they had to put an effort on being assertive in healthcare interactions in order to counteract racism.  -Participants reported the importance of patient-provider racial concordance. |
| Perceptions and experiences regarding the impact of race on the quality of healthcare in southeast Brazil: A qualitative study  Chauhan, A., de Wildt, G., Virmond, M. D. C. L., Kyte, D., Galan, N. G. D. A., Prado, R. B. R., & Shyam-Sundar, V | 2020 | Brazil | To examine the impact of race in healthcare interactions in South east Brazil. | n =19 interviews with healthcare users from various ethnic groups in Brazil. | -Participants felt that racial discrimination might affect access to healthcare for those who were Black but only few participants reported experiencing racism in healthcare. |
| “I was trying to speak to their human side” coping responses of Belgium’s undocumented migrants to barriers in health-care access  Lafaut, D., & Coene, G. | 2020 | Belgium | To explore the coping responses of undocumented migrants when accessing healthcare in Belgium. | n =25 interviews with undocumented migrants in Belgium. | -Participants described a variety of barriers in their access to healthcare, such as dismissive or discriminatory attitudes by healthcare professionals. |
| Caring for indigenous families in the neonatal intensive care unit  Wright, A. L., Ballantyne, M., & Wahoush, O | 2020 | Canada | To explore how indigenous women access neonatal intensive care. | n =19 indigenous mothers. | -Participants viewed nurses as western and dismissive to indigenous culture.  -Participants who had experienced racism or discrimination in the healthcare found it very difficult to trust nurses and healthcare. |
| “I don’t like being stereotyped, I decided I was never going back to the doctor”: Sexual healthcare access among young Latina women in Alabama  Morales-Alemán, M.,M., Gwendolyn, F., & Scarinci, I. C | 2020 | USA | To explore the various barriers Latina women in Alabama have when accessing sexual healthcare. | n =20 Latina women. | -Some participants reported feeling discriminated against because of their ethnicity and reported feeling undeserved and treated with apathy. |
| Listening to Women:  Recommendations from Women of Color to Improve experiences in Pregnancy and Birth Care  Altman MR, McLemore MR, Oseguera T, Lyndon A, Franck LS | 2020 | USA | To explore the recommendations given by women of color to improve healthcare during pregnancy. | n =22 women of color. | -In regards to racism in healthcare, the participants discussed the importance of educating healthcare providers on racism in healthcare.  -Participants also recommended racial concordance with providers. |
| Listening to Women: Understanding and Challenging Systems of Power to Achieve Reproductive Justice in South Carolina.”  Smith, Ellie, Beth Sundstrom, and Cara Delay | 2020 | USA | To explore women’s experiences with reproductive health services in South Carolina using a reproductive justice framework. | n =70 interviews women of various ethnicities living in South Carolina. | -Black women reported experiencing racism in healthcare and described racial bias in healthcare within a larger historical and societal racial structure. |
| "Everything is provided free, but they are still hesitant to access healthcare services": why does the indigenous  community in Attapadi, Kerala continue to experience poor access to healthcare?  George MS, Davey R, Mohanty I, Upton P | 2020 | India | To understand why the indigenous  communities in Attapadi continue to experience poor access to healthcare in spite of both financial protection and adequate coverage of health services. | n =47 indigenous people in Kerala. | -Participants described feeling discriminated against by the health system and described being met with a condescending manner and not taken seriously. |
| Black Pregnant Women "Get the Most Judgment": A Qualitative Study of the Experiences  of Black Women at the Intersection of Race, Gender, and Pregnancy  Mehra R, Boyd LM, Magriples U, Kershaw TS, Ickovics JR, Keene DE | 2020 | USA | To understand Black pregnant  women’s experiences of gendered racism during pregnancy. | n =24 Black pregnant women. | -Participants described how negative assumptions about Black women (e.g. Black women having high infertility) influenced their access to healthcare, quality of accessed healthcare and relationships with healthcare  providers. |
| Implications of institutional racism in the therapeutic itinerary of people with chronic renal failure  Santos Ferreira RB, de Camargo CL, da Silva Barbosa MI, Silva Servo ML, Carneiro Oliveira MM, Leite Leal JA | 2020 | Brazil | To understand the implications of institutional racism in the therapeutic itinerary of patients with chronic renal failure in the search for diagnosis and treatment of the disease. | n =23 people with chronic renal failure | -Differential treatment was reported regarding accessing healthcare including diagnosis of their illness. While White people received diagnosis in outpatient care, Black people were only able to receive diagnosis when hospitalized. |
| Racism in European Health  Care: Structural Violence and Beyond  Hamed S, Thapar-Björkert S, Bradby H, Ahlberg BM | 2020 | Sweden, Portugal and German | To explore experiences of racism in health care by patients in Sweden, Portugal and Germany. | n =11 interviews with patients of migrant background. | -Through the lens of structural violence two processes were identified from the interviews, namely unequal access to resources that led to the silencing of suffering and inequalities in power, which led to the erosion of dignity. |
| Social Determinants of Health and Health Care Delivery: African American  Women’s T2DM Self-Management  Ochieng JM, Crist JD | 2021 | USA | To explore  and describe social determinants of health and health care  delivery that may influence type 2 diabetes management among  African American women. | n =10 African American women with Type 2 diabetes. | -Participants reported inadequate healthcare services related to discrimination. |
| Perceived Structural Racism and Discrimination and Medical Mistrust in the  Health System Influences Participation in HIV Health Services for Black Women  Living in the United States South: A Qualitative, Descriptive Study  Randolph SD, Golin C, Welgus H, Lightfoot AF, Harding CJ, Riggins LF | 2020 | USA | To examine Black women’s perspectives of how perceived structural racism and discrimination, and medical mistrust in the health care  system contribute to their participation in health services. | n =7 focus group discussions (48 women). | -Participants described how providers gave false medical information to patients who were Black.  -Participants discussed systematic inequities towards women in general but also specific challenges in regards to being black.  -Participants talked about how perceived racial bias in healthcare helped women become empowered and advocate for their rights. |
| Community perspectives on the racial disparity in perinatal outcomes  Kalata M, Kalata K, Yen H, Khorshid A, Davis T, Meredith J | 2020 | USA | To explore the conditions that lead to racial perinatal disparities.  To propose practices for addressing racial disparities through  community perspectives. | n =27 African women who have been pregnant previously (6 focus group discussions). | -Women reported healthcare providers’ bias including racial bias as their major concern in accessing care.  -Women felt a lack of power in decision making in healthcare interactions. |
| The journey of aftercare for  Australia's First Nations families whose child had sustained a burn injury: a  qualitative study  Coombes J, Hunter K, Mackean T, Ivers R | 2020 | Australia | To explore barriers and facilitators to culturally safe and appropriate burn aftercare for Australia’s First Nations children. | n =59 First Nation’s family members of children younger than 16 years who have had a burn injury. | -Participants reported experiences of racism and colonialism in healthcare and described feeling disempowered and judged. |
| Racism against Totonaco women in Veracruz: Intercultural competences for health professionals are necessary  Dörr NM, Dietz G | 2020 | Mexico | To explore racism in healthcare towards Totonaco women in Mexico. | Participant observations in medical facilities in Veracruz (2011-2012).  n =60 interviews. | -Indigenous groups have poor access to healthcare.  -Disrespect and dismissal behaviour towards indigenous groups were reported.  -Indigenous groups were also reported to internalize their own oppression. |
| “We get double slammed!”  Healthcare experiences of perceived discrimination among low-income African-  American women  Okoro ON, Hillman LA, Cernasev A | 2020 | USA | To explore the lived experiences of  low-income African-American women in health care interactions. | n =22 in depth interviews with low-income African American women. | -Many participants reported experiences of racism from healthcare professionals.  -Participants reported differential care. They also reported healthcare professionals failing to address and make physical contact with them. |
| What about the men? perinatal experiences of men of color whose partners were at risk for preterm birth, a qualitative study  Edwards, B. N., McLemore, M. R., Baltzell, K., Hodgkin, A., Nunez, O., & Franck, L. S. | 2020 | USA | To explore the experiences of men of color who are partners with women at medical and social risk for preterm birth. | n =12 men. | -Participants identified many barriers to  having a healthy pregnancy and birth. These barriers included discrimination, differential treatment and inadequate support for decision making. |
| Barriers to contraceptive careseeking: The experience of Eritrean asylum-seeking women in Israel  Tsega Gebreyesus, Nora Gottlieb, Zebib Sultan, Habtom Mehari Ghebrezghiabher, Wietse Tol, Peter J Winch, Nadav Davidovitch, Pamela J Surkan | 2020 | Israel | To explore the experiences of Eritrean asylum seekers when seeking contraceptive care in Israel. | n =25 key informants (NGO workers, researchers, community activists and healthcare professionals)  n =12 Eritrean asylum seekers. | -Some Eritrean participants reported experiencing discrimination in healthcare encounters. Participants reported that healthcare providers sometimes used racist terms when addressing them.  -Participants reported that healthcare providers dismissed their healthcare needs and that many hospital required African patients to wear facemasks.  -Although healthcare providers reported discrimination against Eritrean asylum seekers, they attributed the cause of discrimination on communicating difficulties with Eritrean patients. |
| ***Quantitative articles examining healthcare users’ experiences of racism in healthcare (n=41)*** | | | | | |
| **Article title and author/s** | **Year of publication** | **Geographical location** | **Aim of article** | **Methods** | **Key findings in regards to racism in healthcare** |
| Patients’ Beliefs About Racism, Preferences for Physician Race, and Satisfaction With Care.  Chen, F. M., Fryer, G. E. Jr., Phillips, R. L. Jr., Wilson, E., & Pathman, D. E. | 2005 | USA | To examine the association between patients’ beliefs in healthcare racism and use of healthcare services. | n = 1479 Whites, 1,189 African American and 983 Latinos. | -There were stronger beliefs about racial discrimination in healthcare among African Americans.  -Stronger beliefs about racial discrimination in healthcare were associated with preferring an African American physician.  -Only 22% of African Americans preferred an African American physician  -Those who preferred an African American physician and had an African American physician were more likely to rate their physician as excellent compared to African Americans who preferred an African American physician but had a non–African American physician.  -Latinos with stronger beliefs about discrimination in health care were more likely to prefer a Latino physician.  -One third of Latinos preferred a Latino physician. |
| Preference for same-race health care providers and perceptions of interpersonal discrimination in health care  Malat, Jennifer, & Hamilton, M. A. | 2006 | USA | To examine Black Americans’ preferences for Black healthcare providers and its association with racial discrimination in healthcare. | n =1,189 non-Hispanic Black patients. | -The belief that discrimination is frequent in different-race doctor-patient dyads is associated with greater preference for a same-race provider.  -The belief that discrimination occurs regardless of a doctor's race reduces preference for a same-race provider. |
| Patient-reported racial/ethnic healthcare provider discrimination and medication intensification in the Diabetes Study of Northern California (DISTANCE)  Lyles, C. R., Karter, A. J., Young, B. A., Spigner, C., Grembowski, D., Schillinger, D., & Adler, N. | 2011 | USA | To examine whether perceived racial discrimination by healthcare providers is associated with quality of care indicators. | n =10, 409 patients. | -Perceived racial discrimination in healthcare was not associated with medications intensified for hyperglycemia, for hyperlipidemia, for hypertension, and for the composite cohort. |
| Self-reported racial discrimination in healthcare and Diabetes Outcomes  Peek, M. E., Wagner, J., Tang, H., Baker, D. C., & Chin, M. H | 2011 | USA | To examine the association between reported racial discrimination in healthcare and diabetes outcomes. | Data from the Behavioral Risk Factor Surveillance System was used.  *** n *=Unclear* | -Significant associations were found between self-reported healthcare discrimination and most measures of quality of care, and health outcomes related to diabetes, and retinopathy, but not the number of provider foot examinations or diabetes self-management.  -The effects of self-reported discrimination were attenuated or eliminated after controlling for sociodemographics, health status, and access to care. |
| Correlates of patient-reported racial/ethnic health care discrimination in the Diabetes Study of Northern California (DISTANCE  Lyles, C. R., Karter, A.J., Young, B. A., Spigner, C., Grembowski, D., Schillinger | 2011 | USA | To examine possible determinants of self-reported healthcare discrimination. | n =17,795 | -3% percent reported healthcare discrimination.  -Healthcare discrimination was more frequently reported by minorities and those with poorer health literacy, limited English proficiency, and depression. |
| Provider factors and patient-reported healthcare discrimination in the Diabetes Study of California (DISTANCE).  Lyles, C. R., Karter, A. J., Young, B. A., Spigner, C., Grembowski, D., Schillinger, D., & Adler, N. | 2011 | USA | To examine provider-level factors and reported discrimination in healthcare setting. | n = 12,151 patients. | -Patients seeing nurse practitioners as their primary care providers and those rating their provider higher on communication were less likely to report discrimination in healthcare, while those with more visits were more likely to report discrimination.  -Racial concordance was not significant once adjusting for patient race/ethnicity. |
| Perceived racism, medication adherence, and hospital admission in African-Caribbean patients with psychosis in the United Kingdom  Chakraborty, A., King, M., Leavey, G., & McKenzie, K | 2011 | UK | To examine whether there is a link between perceived racism in healthcare and medical adherence. | n = 100 patients  Patients were asked about racism at baseline with perceived racism scale. Adherence to medicine as well as hospital admission data were measured. | -There was associations between total perceived racism for the previous year, lifetime racism, and everyday racism for the previous year with subsequent medication adherence.  -Shame felt about health system racism was associated with increased adherence.  -Powerlessness health system racism was associated with fewer subsequent hospital bed days.  -Health system racism was associated with both the number of subsequent hospital bed days, and admission length.  -Stratified analyses showed that both baseline adherence and 6-month estimated adherence appeared to mediate these effects. |
| Socially-Assigned Race, Healthcare Discrimination and Preventive Healthcare Services  MacIntosh, T., Desai, M. M., Lewis, T. T., Jones, B. A., & Nunez-Smith, M | 2013 | USA | To examine the associations between socially-assigned race and healthcare discrimination and receipt of selected preventive services. | n  = 6,837 Minority/Minority  n = 929 Minority/White  n = 25,913White/White.  Measures included reported healthcare discrimination and receipt of vaccinations and cancer screenings. | -Racial/ethnic minorities who reported being socially-assigned as white were more likely to receive preventive vaccinations and less likely to report healthcare discrimination compared with those who are socially-assigned as minority. |
| Discrimination attributed to mental illness or race-ethnicity by users of community psychiatric services  Jheanell Gabbidon, Simone Farrelly, Stephani L Hatch, Claire Henderson, Paul Williams, Dinesh Bhugra, Lisa Dockery, Francesca Lassman, Graham Thornicroft, Sarah Clement | 2014 | UK | To examine participants’ experienced discrimination. | n =202 service users with severe mental health illnesses.  The major experiences of discrimination scale was used. | -88% of participants reported discrimination in at least one life domain.  -The most common area of major discrimination was healthcare (44%).  -The most common attributions for major discrimination were mental illness (57%) and race-ethnicity (24%).  -Participants in the Black group were most likely to endorse race-ethnicity as a main attribution for discrimination. |
| Perceived Discrimination in Health Care is Associated with a Greater Burden of Pain in Sickle Cell Disease  Carlton Haywood Jr, Marie Diener-West, John Strouse, C Patrick Carroll, Shawn Bediako, Sophie Lanzkron, Jennifer Haythornthwaite, Gladys Onojobi, Mary Catherine Beach | 2014 | USA | To describe the extent to which patients with sickle cell disease perceive discrimination from healthcare providers, and to examine the association of these experiences with the burden of chronic sickle cell disease pain. | n =291 African American patients with sickle cell disease.  Measurements of discrimination from healthcare providers were analysed. | -Higher burden due to discrimination from healthcare providers was reported in this study compared to other studies including greater amount of disease-based versus race-based discrimination.  -Age and having difficulty persuading providers about pain were independently associated with race-based discrimination.  -Older age, greater emergency room utilization, having difficulty persuading providers about pain, daily chronic pain, fewer “good days” during a week, and a higher severity of pain on their “good days” were independently associated with greater disease-based discrimination. |
| Perceived racial discrimination in health care, completion of standard diabetes services, and diabetes control among a sample of American Indian women  Gonzales, K. L., Lambert, W. E., Fu, R., Jacob, M., & Harding, A. K. | 2014 | USA | To examine perceived experiences of racial discrimination in healthcare and its associations with completing standards of care for diabetes management and diabetes control. | n =200 adult American Indian women with type 2 diabetes from 4 healthcare facilities in reservations. | -67% of American Indian women reported discrimination during their lifetime of health care.  -After adjusting for patient characteristics, perceived discrimination was significantly associated with lower rates of dental exam; checks for blood pressure, creatinine, and total cholesterol; and pneumococcal vaccination.  -The association between perceived discrimination and total number of diabetes services completed was not statistically significant.  - Perceived discrimination in healthcare was associated with having A1C values above target levels for diabetes control in unadjusted and adjusted models, but no association was observed for blood pressure or total cholesterol. |
| A Study of Perceived Racial Discrimination in Black Men Who Have Sex with Men (MSM) and its Association with Healthcare Utilization and HIV Testing  Irvin, R., Wilton, L., Scott, H., Beauchamp, G., Wang, L., Betancourt, J., … Buchbinder, S. | 2014 | USA | To examine the association of healthcare racial discrimination with healthcare utilization and HIV testing among HIV negative patients. | n =1167 HIV negative Black men who have sex with men. | -Racial discrimination in healthcare was positively associated with seeing a provider and HIV testing. This suggests that barriers other than racial discrimination may be driving health disparities related to access to medical care and HIV testing among Black men who have sex with men. |
| Patient-reported Communication Quality and Perceived Discrimination in Maternity Care  Attanasio, L., & Kozhimannil, K. B | 2015 | USA | To examine racial ethnic disparities in patient-reported communication.  To report perceived discrimination in healthcare. | n =2400  Data was used from the Listening to Mothers III survey, a national sample of women who gave birth to a singleton baby in a US hospital. | -Over 40% of women reported communication problems in prenatal care.  -24% perceived discrimination during their hospitalization for birth.  -Having hypertension or diabetes was associated with higher odds of reporting each type of perceived discrimination.  -Black and Hispanic (vs. White) women had higher odds of perceived discrimination due to race/ethnicity. |
| Foreign-Born Latinos Living in Rural Areas are more likely to Experience Health Care Discrimination: Results from Proyecto de Salud para Latinos  López-Cevallos, D. F., & Harvey, S. M | 2016 | USA | To examine the association between immigration status and perceived health care discrimination among Latinos living in rural areas. | n =349 adult Latinos living in rural areas. | -39.5% of the participants experienced healthcare discrimination.  - Discrimination was higher among foreign-born (44.9 %) rather than US-born Latinos (31.9 %).  -Foreign-born Latinos were significantly more likely to experience health care discrimination, even after controlling for other relevant factors. |
| Dimensions of Racial Identity and Perceived Discrimination in Health Care  Stepanikova, I., & Oates, G. R | 2016 | USA | To examine the relationships between perceived discrimination in healthcare and self-identified race/ethnicity and perceived attributed race/ethnicity. | Data on discrimination in healthcare from the Behavioral risk factor surveillance system data from 2004-2013.  *n =Unclear* | -Both dimensions of racial/ethnicity healthcare contributed independently to perceived discrimination in healthcare.  - After controlling for self-identified race/ethnicity, respondents who reported being classified as Black, Asian, Hispanic, and Native American had higher likelihood of perceived discrimination than respondents who reported being classified as White.  -After taking perceived attributed race/ethnicity into account, self-identified Blacks, Native Americans, and multiracial respondents were more likely to report perceived discrimination than counterparts who self-identified as White. |
| Perceived Discrimination and Privilege in Health Care: The Role of Socioeconomic Status and Race  Stepanikova, I., & Oates, G. R. | 2017 | USA | To examine the association between racial privilege and racial discrimination in healthcare varied with race and socio-economic status. | Behavioral Risk Factor Surveillance System data was used.  *n =Unclear* | -Perceptions of racial privilege were less common among blacks and Native Americans compared with whites.  -Perceptions of racial discrimination were more common among minorities.  -In Whites, higher income and education contributed to increased perceptions of privileged treatment and decreased perceptions of discrimination.  -Blacks reported more discrimination and less privilege at higher income and education levels.  -Across racial groups, respondents who reported foregone medical care due to cost had higher risk of perceived racial discrimination.  -Health insurance contributed to less perceived racial discrimination and more perceived privilege only among Whites. |
| Racial and cultural minority experiences and perceptions of health care provision in a mid-western region.  Shepherd, S. M., Willis-Esqueda, C., Paradies, Y., Sivasubramaniam, D., Sherwood, J., & Brockie, T | 2018 | USA | To explore the self-reported healthcare experiences for racial and cultural minority Americans. | n =117 racial and cultural minority Americans | -Racial/cultural minority groups (African Americans, Native Americans, Latino/a Americans, and Asian Americans) reported general satisfaction with current healthcare providers, low levels of both healthcare provider racism and poor treatment, high levels of cultural strength and good access to health care services.  -Native American participants however, reported more frequent episodes of poor treatment compared to other groups.  -Poor treatment predicted lower levels of treatment satisfaction and racist experiences predicted being afraid of attending conventional health care services.  -Cultural strength predicted a preference for consulting a healthcare professional from the same cultural background. |
| Factors affecting trust in healthcare among middle-aged to older Korean American women  Hong, H. C., Lee, H., Collins, E. G., Park, C., Quinn, L., & Ferrans, C. E | 2018 | USA | To examine factors influencing trust in the healthcare system and trust in providers among Korean American women. | n =168 Korean American women. | -Acculturation was positively related to trust in healthcare providers.  -Discrimination in the healthcare system was inversely related to trust in healthcare providers.  -Discrimination in healthcare was positively related to distrust in healthcare system.  -Other factors such as length of stay in the US was inversely related to distrust in the healthcare system.  -Trust in healthcare providers and distrust in the healthcare system were moderately correlated. |
| Declined care and discrimination during the childbirth hospitalization  Attanasio LB, Hardeman RR | 2019 | USA  Quantitative | To examine the relationship between declining procedures and discrimination during the childbirth hospitalization. | n = 2,400 women age 18–45 who gave birth to a singleton baby in a U.S. hospital in (2011–2012). | -Women who reported having declined care for themselves or their infant during childbirth hospitalization were more likely to report “poor treatment” based on race and ethnicity, insurance status or having a difference of opinion with a healthcare provider.  -Significantly larger magnitude of Black women perceived discrimination due to difference of opinion with a healthcare provider compared to White women. |
| Racial Centrality May Be Linked to Mistrust in Healthcare Institutions for African Americans.”  Cuevas, Adolfo G., and Kerth O’Brien | 2019 | USA | To examine African American’s racial identity’s association with mistrust in healthcare. | n =220 African Americans. | -Racial identity was positively associated with medical mistrust and with increased experience of racism in healthcare but not with mistrust in physicians. |
| Racial discrimination and uptake of dental services among American adults  Sabbah, W., Gireesh, A., Chari, M., Delgado-Angulo, E. K., & Bernabé, E | 2019 | USA | To examine the relationship between racial discrimination and the use of dental services. | n =464,664 healthcare users from the CDC telephone survey, 2014. | -Dental visits were less common among those who reported experiencing racism in healthcare and those who reported emotional impact due to discrimination. |
| “Perceived Microaggressions in Health Care: A Measurement Study  Cruz, Daniel, Yubelky Rodriguez, and Christina Mastropaolo | 2019 | USA | To examine the psychometric properties of the Microaggressions in Health  Care Scale including factor structure, measurement invariance, and internal consistency reliability. | n =296 African American and Latino  Participants. | -Many participants experienced negative stereotypes from doctors about their racial/cultural groups.  -Participants reported that physicians were culturally insensitive and/or that they avoided addressing diversity in their medical encounters. |
| Patient-clinician interactions and disparities in breast cancer care: the equality in breast  cancer care study  Gonzales FA, Sangaramoorthy M, Dwyer LA, Shariff-Marco S, Allen AM, Kurian AW, Yang J, Langer MM, Allen L, Reeve BB, Taplin SH, Gomez SL | 2019 | USA | To examine whether interpersonal aspects of patient-clinician interactions, such as patient-perceived medical discrimination, clinician mistrust, and treatment decision-making contribute to racial/ethnic/educational disparities in breast cancer care. | n =542 Asian/Pacific Islander, Black, Hispanic, and White women identified through the Greater Bay Area Cancer Registry, ages 20 and older diagnosed with a first primary invasive breast cancer. | -Non-college-educated Black women had lower odds of guideline-concordant care vs. college-educated White women.  -Odds of excellent perceived quality of care were lower among college-educated Hispanic women and Asian Pacific Islander women regardless of education vs. college-educated White women, women reporting low and moderate levels of discrimination vs. none, and women reporting any clinician mistrust vs. none.  -Disparities in guideline-concordant care and perceived quality of care persisted after controlling for medical discrimination, clinician mistrust, and decision-making. |
| Changes in Perceptions  of Discrimination in Health Care in California, 2003 to 2017  Schulson LB, Paasche-Orlow MK, Xuan Z, Fernandez A | 2019 | USA | To determine whether perceptions of discrimination in healthcare has changed in California from 2003-2017. | n = 84 088 participants in 2003 to 2005 and 63 242 participants in 2015 to 2017 in California from various ethnicities. | -Reports of recent discrimination in healthcare in California decreased substantially in 2015 to 2017 compared with 2003 to 2005 for Latino individuals, immigrants, and people with limited English proficiency but not for African American individuals. |
| Experience of racism and associations with unmet need and healthcare satisfaction: the 2011/12 adult New Zealand Health Survey  Harris, R. B., Cormack, D. M., & Stanley, J. | 2019 | New Zealand | To examine associations between experiences of racism and unmet need ad healthcare satisfaction. | n=12,596 adults were taken from the New Zealand Health Survey. | -Maori, Pacific and Asian groups experienced more racism by a health professional as well as other forms of racism compared to Europeans.  -Both racism measures were associated with higher unmet need in healthcare and lower satisfaction with a usual medical centre. |
| Perceived discrimination in medical settings and  perceived quality of care: A population-based study in Chicago  Benjamins MR, Middleton M | 2019 | USA | To examine the association between perceived discrimination in healthcare and quality of care. | n = 1,543  Black, White, Mexican, Puerto Rican, and other adults in 2015-2016. | -Individuals reporting discrimination had more than twice the odds of reporting fair or poor quality of care.  -Perceived discrimination in medical settings was significantly associated with report of not having enough time with the physician and not being as involved in decision-making as desired. |
| Health care experiences and birth outcomes: Results of an Aboriginal birth Cohort  Brown SJ, Gartland D, Weetra D, Leane C, Francis T, Mitchell A, Glover K | 2019 | Australia | -To examine the relationships between perceived discrimination in perinatal care and birth outcomes of women giving birth to an Aboriginal baby in Australia. | n = 344 women living in urban, regional and remote areas of South Australia. | -51% of the women perceived that they had experienced discrimination or unfair treatment in healthcare.  - Women experiencing three or more stressful events or social health issues were more likely to perceive that care was discriminatory or unfair.  -Women who perceived that they had experienced discrimination in perinatal care were more likely to have a baby with a low birth weight. |
| Discrimination in the United States: Experiences of Asian Americans.  McMurtry CL, Findling MG, Casey LS, Blendon RJ, Benson JM, Sayde JM, Miller C. | 2019 | USA | To examine experiences of racial discrimination among Asian Americans. | n =500 Asians and 902 White USA adults from 2017. | -3% percent of Asians reported discrimination in healthcare encounters.  -In unadjusted models, East and South Asians were more likely than Whites to report experiences of institutional discrimination. |
| Discrimination in the United States: Experiences of Latinos  Findling MG, Bleich SN, Casey LS, Blendon RJ, Benson JM, Sayde JM, Miller C | 2019 | USA | To examine experiences of racial discrimination among Latinos in the USA. | n = 803 non-Hispanic White adults from 2017. | -20% reported experiencing discrimination in clinical encounters.  -17% avoided seeking healthcare for themselves or family members due to anticipated discrimination. |
| Discrimination in the United States: Experiences of black  Americans.  Bleich SN, Findling MG, Casey LS, Blendon RJ, Benson JM, SteelFisher GK, Sayde JM, Miller C | 2019 | USA | To examine the experiences of racial discrimination among blacks in the USA (including racism in healthcare). | n =802 non-Hispanic blacks. n = 902 non-Hispanic White USA adults from a telephone survey conducted in 2017. | -32% of blacks reported experiencing racism in clinical encounters.  -22% avoided seeking care due to fear of racism in clinical encounters. |
| Discrimination in the United States: Experiences of Native  Americans  Findling MG, Casey LS, Fryberg SA, Hafner S, Blendon RJ, Benson JM, Sayde  JM, Miller C | 2019 | USA | To examine the experiences of racial discrimination (including racism in healthcare) among Native Americans in the USA. | n = 42 Native American  n =902 White USA adults from a telephone survey conducted in 2017. | -23% of Native Americans reported experiencing racism in clinical encounters.  -15% avoided seeking healthcare for themselves or family members due to anticipated discrimination in healthcare. |
| Impact of Perceived Racism on  Healthcare Access Among Older Minority Adults  Rhee TG, Marottoli RA, Van Ness PH, Levy BR | 2019 | USA | To investigate whether perceived racism is associated with delayed care among minority older groups and whether this relationship is medicated by poor doctor communication. | n =1,756 minority people 65 years old or older. Data was obtained from the California telephone survey 2017-2018 | -Perceived racism in healthcare was associated with greater odds for delayed care.  -Perceived racism in healthcare was associated with greater odds of poor doctor communication.  -Poor doctor communication mediated the association between perceived racism and delayed care. |
| Mistrust, Racism, and Delays in Preventive Health Screening Among African-American Men  Powell W, Richmond J, Mohottige D, Yen I, Joslyn A, Corbie-Smith G. Medical | 2019 | USA | To examine the associations between medical mistrust, perceived racism in healthcare, everyday racism, and preventive health screening delays. | n =610  Data from the African-American Men’s Health and Social Life study from 2003-2009. | -Higher mistrust among the informants was associated with delays in routine health visits.  -Higher level of perceived racism in healthcare was associated with the increased likelihood of delaying cholesterol screening visits. |
| Contested and mistreated? contested racial identities and unfair treatment due to race.  Farrell, A. | 2020 | USA | To examine the association of contested racial identities and racism in healthcare. | n = 132,436 from the 2004–2010 Behavioural Risk Factor  Surveillance System. | -Identity contestation is associated with the likelihood of perceiving unfair treatment in healthcare.  -Contestation is associated with a greater likelihood of reporting unfair treatment due to race for Whites but a lower likelihood of reporting unfair treatment for Latinx and Black individuals. |
| Unmet health needs and discrimination by  healthcare providers among an Indigenous population in Toronto, Canada  Kitching GT, Firestone M, Schei B, Wolfe S, Bourgeois C, O'Campo P, Rotondi M, Nisenbaum R, Maddox R, Smylie J | 2020 | Canada | To examine the association between experience of discrimination by healthcare providers and having unmet health needs within the Indigenous population of Toronto. | n = 917 self-identified Indigenous adults within Toronto. | -Discrimination by a healthcare provider was positively associated with unmet health needs among participants. |
| Depression Treatment Preferences by Race/Ethnicity and Gender and Associations between Past Healthcare Discrimination Experiences and Present Preferences in a Nationally Representative Sample  Sonik, Rajan Anthony, Timothy B. Creedon, Ana Maria Progovac, Nicholas Carson, Jonathan Delman, Deborah Delman, and Benjamin Lê Cook | 2020 | USA | To assess depression treatment preferences by race/ethnicity and  gender in a representative community sample. | n = 711 adults with moderate to severe depression from the nationally representative online panel in 2017 | - Experience of racism in healthcare was associated with significantly lower preferences for talk therapy and greater preferences for medication among non-Hispanic Black respondents and women. |
| Is racial discrimination associated with number of missing teeth among American adults?  Muralikrishnan, M., & Sabbah, W. | 2020 | USA | To assess the association of racial discrimination with tooth loss among American adults. | n = 5852 from 2014. | -Among those reporting discrimination at a healthcare facility, there was 141% increase in tooth loss compared to those not reporting discrimination. |
| Insurance Types, Usual Sources of Health Care, and Perceived Discrimination.  Alcalá HE, Ng AE, Gayen S, Ortega AN | 2020 | USA | To understand the factors associated with experiences of discrimination  in healthcare. | n = 63,100  Data from the 2015 to 2017 California Health Interview. | -Insurance type and sources of care are associated with lifetime perceptions of discrimination in health care. |
| Association of sociocultural factors with initiation of the kidney transplant evaluation  process.  Hamoda RE, McPherson LJ, Lipford K, Jacob Arriola K, Plantinga L, Gander JC,  Hartmann E, Mulloy L, Zayas CF, Lee KN, Pastan SO, Patzer RE | 2020 | USA | To examine the associations between sociocultural factors and initiating kidney transplant evaluation. | n = 528 patients with end‐stage renal disease from 2014‐2016. | -Medical mistrust, patient perceived racism in healthcare and experienced discrimination in health care were significantly associated with not initiating the kidney transplant evaluation. |
| Discrimination in healthcare as a barrier to care:  experiences of socially disadvantaged populations in France from a nationally  representative survey  Rivenbark JG, Ichou M | 2020 | France | To examine the experiences of healthcare discrimination in France. | n = 21,761 people with an oversampling of immigrants. | -Reports of discrimination in healthcare were higher among women, immigrants from Africa or Overseas France as well as Muslims.  -Experiences of discrimination among the above mentioned groups explained significant proportion of their disparity in foregone care. |
| Effect of Physician Gender and  Race on Simulated Patients' Ratings and Confidence in Their Physicians: A Randomized Trial  Solnick RE, Peyton K, Kraft-Todd G, Safdar B | 2020 | USA | To examine whether physician gender and race affect participant ratings in scenarios in which physician competence is challenged. | n = 3592 of geographically diverse sample. | -Simulated physician gender and race did not significantly affect participant satisfaction or confidence in physician clinical judgment compared with a White male physician control. |
| ***Mixed method articles examining healthcare users’ experiences of racism in healthcare (n=7)*** | | | | | |
| **Article title and author/s** | **Year of Publication** | **Geographical location** | **Aim of article** | **Methods** | **Key findings in regards to racism in healthcare** |
| Primary health care experiences of Hispanics with serious mental illness: a mixed-methods study  Cabassa, L. J., Gomes, A. P., Meyreles, Q., Capitelli, L., Younge, R., Dragatsi, D., … Lewis-Fernández, R | 2014 | USA | To explore healthcare experiences of Hispanics with serious mental health illness. | *Quantitative*  Structured patient interviews  medical chart  abstractions  n =40. Perceived discrimination and stigma was measured.  *Qualitative*  n =24 Hispanic healthcare users. | -Three-quarters of participants reported that racism was a problem or major problem in the USA health care system.  -Qualitative data revealed that participants experienced racism in the form of dismissal of symptoms by healthcare providers. –  -Participants also experienced more blatant racist remarks from healthcare providers who did not take them seriously. Racism is complicated by the discrimination which participants experiences due to their mental health illness. |
| Health Care Use and Barriers to Care among Latino Immigrants in a New Migration Area  Jacquez, F., Vaughn, L., Zhen-Duan, J., & Graham, C. | 2016 | USA | To understand the experiences of Latino immigrants in a specific context in USA where there is increased Latino population. | *Quantitative*  n =516 Latino immigrants.  *Qualitative*  (Focus group discussion)  n =34 Latino immigrants. | -Participants identified the following barriers to healthcare: language issues, lack of interpreters and long waiting time.  -Participants also reported experiencing racism which made them feel apprehensive when seeking healthcare. Racism was described in providers asking offensive questions, and assuming that the participants were less knowledgeable and providing lower quality of care compared to Non-Latinos. |
| Measuring racial microaggression in medical practice  Almond AL | 2019 | USA | To validate the Racial Microaggression in Counseling Scale when the term ‘therapist’ was replaced with ‘physician’. | *Quantitative and qualitative*:  n =91 racial minorities | -The scale was related to the racial incongruence between patient and provider.  -Qualitative findings support the original concepts and themes used when developing the 10-item measure in a counselling setting. |
| Ethnic discordance: Why do some patients prefer to be treated by physicians from other ethnic groups?  Keshet Y | 2019 | Israel | To evaluate the rates of Jewish and Arab patients who prefer patient-provider.  To examine the reasons for patients’ preference. | *Quantitative*  n =760 (505 Jews, 255 Arabs).  *Qualitative*  n =38 Jewish and Arab patients in Israel. | -Survey's findings indicate that Arabs are more likely to prefer to be treated by a Jewish physician than are Jews to prefer an Arab physician.  -The reason for this preference by Arab patients is internalised racism. The reason for the preference of Jewish patients to Arab healthcare professionals is that the latter are perceived as more professional due to their need to prove themselves. |
| Mental health care among blacks in America: Confronting racism and constructing solutions  [Sirry M. Alang](https://www-ncbi-nlm-nih-gov.ezproxy.its.uu.se/pubmed/?term=Alang%20SM%5BAuthor%5D&cauthor=true&cauthor_uid=30687928) | 2019 | USA | To describe reasons for unmet need for mental healthcare among blacks. | *Quantitative*  National Survey on Drug Use and Health (2011‐2015 )  n = 1237  *Qualitative*  Focus group discussion  n =30 | -Higher education was associated with greater odds of reporting stigma and minimization of symptoms as reasons for unmet need.  - Racism in healthcare according to the focus group discussion, causes mistrust in mental health service systems. |
| Aboriginal patient and interpreter perspectives on the delivery of culturally safe hospital-based care  Mithen, V., Kerrigan, V., Dhurrkay, G., Morgan, T., Keilor, N., Castillon, C., . . . Ralph, A. P. | 2020 | Australia | To validate an experience of care survey and document advice from Aboriginal interpreters in healthcare interactions. | n =68 medical charts for aboriginal patients from April to August 2018 + n =73 patient experience surveys + n =4 interviews with interpreters | -Prominent themes were loneliness, homesickness, problems with the hospital physical environment, racism and a lack of staff understanding of patients’ complaints.  -Key needs articulated by patients were better social and emotional support in hospital to combat loneliness; greater respect amongst staff for patients' cultural obligations and freedom from racism. |
| Identifying healthcare  experiences associated with perceptions of racial/ethnic discrimination among  veterans with pain: A cross-sectional mixed methods survey  Hausmann LRM, Jones AL, McInnes SE, Zickmund SL | 2020 | USA | To identify specific domains of dissatisfaction that are associated with racial/ethnic-based perceived discrimination while seeking healthcare among White, African American, and Latino veteran patients with pain. | *Quantitative*  n = 622 participants.  *Qualitative*  n =10 telephone surveys  n =90 veterans from different medical facilities and of various ethnicities | -622 participants (30.4% White, 37.8% African American, 31.8% Latino; 57.4%  female; mean age = 53.4) reported a median discrimination score of 1.0.  -233 (37.5%) perceived any racial/ethnic discrimination in healthcare.  -7 of 10 qualitative domains were significantly associated with perceived discrimination: dissatisfaction with care quality, facilities, continuity of care, interactions with staff, staff demeanor, unresolved pain, and pharmacy services |
| ***Qualitative articles examining healthcare staff’s experiences of racism in healthcare (n=23)*** | | | | | |
| **Article title and author/s** | **Year of publication** | **Geographical location** | **Aim of study** | **Methods** | **Key finding in regards to racism in healthcare** |
| Does race still matter in nursing? The narratives of African-American nursing faculty members  Beard, K. V., & Julion, W. A. | 2001 | USA | To explore the narratives of African American medical setting members on their views on racism in nursing. | n =23 African American medical setting members. | -Many of the participants discussed their concerns on racism and its effect on the working environment.  -Participants felt that their academic credibility was questioned and that they were socially marginalised.  -Participants also stated that faculty members and students attributed their academic success to being a minority instead of personal merits and hard work.  -Many participants demonstrated resilience in face of racism. Participants considered racism to be everywhere and thus one had to keep working against racism and disrespectful students. |
| Perceived needs of African-American caregivers of elders with dementia  Lampley-Dallas, V. T., Mold, J. W., & Flori, D. E. | 2001 | USA | To examine caregivers perception of experience of residents in regards to racism in healthcare. | n =13 African American caregivers. | -African American caregivers were frustrated with healthcare and service providers. They perceived racism as absence of local support group. |
| Immigrant nurses’ experience of racism  Hagey, R., Choudhry, U., Guruge, S., Turrittin, J., Collins, E., & Lee, R. | 2001 | Canada | To explore immigrants nurses’ experience of racism | n =9 immigrant nurses | -Nurses reported being marginalised and subjected to overt racism from managers which escalated leading them to file complaints. However, their complaints were dismissed and they were framed as problem makers.  -These experienced led to nurses experiencing stress and physical and mental health issues such as depression and increased blood pressure. |
| Health disparities, social injustice, and the culture of nursing  Giddings, L. S. | 2005 | New Zealand and USA | To explore healthcare professionals experience with racism in healthcare settings. | n = 25 nurses from various ethnicities from both the USA and New Zealand (United States (n = 13) and Aotearoa New Zealand (n = 13). | -Nurses perceived that they were homogenized by other staff members and had difficulties in being promoted.  -Main stream nursing lacked awareness on racism.  -Majority nurses were punished if they stood by minority nurses (checked upon, given more night shifts and given more chores).  -To try to survive, nurses of color tried to fit in and deny their ethnicity and lived in two worlds and in contradiction |
| Is it racism? Skepticism and resistance towards ethnic minority care workers among older care recipients  Jönson, H | 2007 | Sweden | To explore how healthcare professionals view racism. | n =12 care givers were interviewed | -Racism was seen as a sensitive issue by the participants.  -Racism was viewed as rare but does exist as described by the majority group who framed racism as mostly about fear of the unknown. In contrast care givers with a minority background described the issue as frequent.  -Ethnic minority care givers endured care recipients negative complaints about migrants. These negative view included viewing migrants as exploiting the welfare system, committing crimes, taking jobs from Swedes, being unclean, ungrateful and should be sent back to where they came from.  -Issues with racism were reported to disappear once the care giver and client got to know each other.  -Complaints about language in regards to ethnic minority’s language abilities was also reported by managers.  -All representatives embraced the official norm of anti-racism. |
| Embodiment of discrimination and overseas nurses’ career progression.  Larsen, J. A | 2007 | UK | To explore overseas nurses experience of discrimination within healthcare. | The study was part of a larger project. Two cases were used in this study. | -Discrimination was reported to be either blatant or subtle as aversive racism.  -Discrimination was both interpersonal and at the institutional level and is a form of symbolic violence.  -Discrimination could be internalised and affects nurses’ habitus and ability for professional development. |
| Overseas nurses’ experiences of discrimination: a case of racist bullying?  Allan, H. T., Cowie, H., & Smith, P. | 2009 | UK | To examine how healthcare providers experience racism in the National Health System. | Three case studies of nurses as part of a larger national study. | -Experience of both overt and covert racism and discrimination in the workplace were reported.  -Abusive use of power my managers were described which led to exclusion from the ward team and in one case being fired.  -Nurses responded by being firm, recording bullying evidence as well as lower self-esteem in one case. |
| Experiences of racism and discrimination among migrant care workers in England: findings from a mixed-methods research project.  Martin Stevens, Shereen Hussein, Jill Manthorpe | 2011 | UK | To report on the experiences of migrant care workers in their work places. | n =26 interviews with employers. n =12 interviews with human resources managers.  n =96 migrant workers.  n =27 UK frontline workers in the public and independent sectors.  n =35 people using services. n =6 carers in each research site. n =18 national sample of refugees and asylum seekers. n =5 representatives of organizations supporting refugees. | -Cultural differences contributed to some communication issues between workers and residents were reported.  -Many migrant workers experienced bullying from managers and co-workers and over one third experienced racism. This was mostly experienced by Black Africans.  -Racism was often seen to underlie the experiences of bullying.  -Racism was also experienced from residents which the care workers explained as due to old age. |
| Black African nurses’ experiences of equality, racism, and discrimination in the National Health Service  Likupe, G., & Archibong, U | 2013 | UK | To explore how healthcare providers experienced racism in National Health System from co-workers and patients. | n = 30 Black African nurses. | -Racism was experienced from both White nurses and other overseas nurses.  -Nurses lost confidence in their abilities because they were told they were not good enough and perceived as arrogant if they voiced their dismay.  -Nurses experienced racism from patients and relatives, in job opportunities and professional development. |
| Hispanic nurses’ experiences of bias in the workplace  Moceri, J. T. | 2014 | USA | To explore the experiences of minority healthcare providers of racism in healthcare. | n = 111 Hispanic nurses participated and provided written opinions on bias. | **-**Racism from patients was tolerated because they were sick or because the nurses did not expect the management to deal with it.  -Institutional racism and racism from other staff were described as being overlooked and undervalued as well as needing to prove competency. This was described as more difficult to deal with than the bias from patients due to its effect on career development. |
| Filipina nurses’ transition into the US hospital system  Lin, L.-C. | 2014 | USA | To examine the experiences of minority healthcare providers of racism in healthcare. | n =31 Filipina nurses. | -Various levels of adaptation were reported: early adaptation to the USA system and late adaptation. During late adaptation nurses stated that they experienced racism in healthcare. |
| Managing for quality aged residential care with a migrant workforce  Ngocha-Chaderopa, N. E., & Boon, B | 2016 | New Zealand | To explore how managers of aged residential care facilities work to ensure delivery of quality care through their migrant care workers. | n =16 managers of care homes. | -Managers of migrant care workers reported experiencing language and communication issues but also reported experiences of racism from residents.  -Migrant care workers were expected to be patient when experiencing racism, ignore their experiences of racism and keep working. However, other strategies were employed including introducing the carers to the residents as soon as they start and defending the carer. |
| Healthcare Providers' Formative Experiences with Race and Black Male Patients in Urban Hospital Environments.  MV Plaisime | 2017 | USA | To assess healthcare providers’ perception of race and Black patients in medical interactions. | n =16 semi structured interviews with healthcare providers (both Black and non-Black providers). | -Both Black and non-Black providers described Black men as scary, unreliable, less educated and less compliant.  -Providers described incidents of racism in healthcare by other providers towards Black patients. |
| Understanding Mental Health: What Are the Issues for Black and Ethnic Minority Students at University?  Arday, J | 2017 | UK | To assess the barriers in accessing culturally appropriate mental health services for Black and minority ethnic students. | n =32 Black and Minority Ethnic students at a medical educational setting. | -Participants talked about the difficulties of navigating racism and how this grinds their mental health. This difficulty was coupled with the inability of healthcare professionals to understand their experiences of racism.  -Participants also reported on differential treatment on mental health issues between them and White counterparts. |
| “I Can Never Be Too Comfortable”: Race, Gender, and Emotion at the Hospital Bedside  Cottingham, M. D., Johnson, A. H., & Erickson, R. J | 2018 | USA | To examine how race and gender shape nurses' emotion practice. | n =48 nurses. | -Racism from patients and from co-workers, both subtle and direct was reported.  -Racism from patients resulted in emotional labour and negotiation with patients. This led to job related stress and depleted emotional resources that influenced patient care. |
| Race-based experiences of ethnic minority health professionals: Arab physicians and nurses in Israeli public healthcare organizations  Keshet, Y., & Popper-Giveon, A | 2018 | Israel | To examine healthcare providers experiences of racism within healthcare setting. | n =10 Arab physicians and 10 Arab nurses. | -Blatant racism was mostly experienced by nurses from patients. Patients refused to be treated by Arabs. Verbal abuse and use of physical violence was also reported. These experiences left the nurses afraid and rejected. These experiences were affected by the ongoing Israeli-Arab conflict.  - When violence was used security was called but when racism was not articulated as physical violence, as in patients refusing treatment, someone else was called to treat the patient.  -Racism from other staff members was also reported. This was mostly impacted by the national conflict.  -Physicians felt that they had to prove themselves at every level which impacted their career development. They dealt with this by working harder than other doctors. |
| The Secret Drama at the Patient’s Bedside-Refusal of Treatment Because of the Practitioner’s Ethnic Identity: The Medical Staff ’s Point of View  Popper-Giveon, A., & Keshet, Y. | 2018 | Israel | To examine healthcare providers experiences of racism within healthcare setting. | n =50 interviews with 10 managers and 40 Jewish and Arab healthcare professionals. | -The hospital was perceived as egalitarian. However incidents of treatment refusal by patients were reported. These were reported more by nurses and students and not as much by physicians. All cases but two was refusal from Jewish patients to be treated by Arab healthcare professionals.  -Most incidents of patients refusal was initiated by relatives and not the patient.  -National conflict escalated incidents of patient refusal.  -Healthcare professionals who were rejected by patients felt humiliation and insult which they carried with them a long time.  -Two responses to patient refusal were reported; changing the practitioner as desired by the patient or refusing to change the practitioner whereby patients either accepted treatment or went somewhere else. The latter was not often employed due to ethical dilemmas. |
| Primary Care Clinician and Clinic Director Experiences of Professional Bias, Harassment, and Discrimination in an Underserved Agricultural  Region of California  Ko M, Dorri A | 2019 | USA | To describe the professional experiences of a diverse group of primary care clinicians and clinic directors in an underserved rural and agricultural region of California. | n =26 diverse group of healthcare professionals in primary care settings. | -Many participants reported experiences of bias, harassment, and discrimination based on gender, race/ethnicity, and/or sexual orientation and gender identity.  -Some participants experienced fatigue due to racism in healthcare both from patients and other healthcare professionals |
| The Experience and Motivations of Midwives of Color in Minnesota: Nothing for Us Without Us  Almanza J, Karbeah J, Kozhimannil KB, Hardeman R | 2019 | USA | To examine the lived experience and knowledge of midwives of color associated with an African American‐owned community birth center in Minnesota. | n =7  Individual interviews midwives of color. | -Midwives expressed that providing care to racially or culturally similar families was important and motivating to them. |
| African American Nurses Describe Experiences of Mistrust and Trust While in Nursing School  White BJ, Mentag NM, Kaunda BR | 2020 | USA | To explore the experiences of African American nurses in nursing school. | n =14 African nursing students. | -Participants reported mistrust from European American nursing students and prevalent negative stereotypes about African Americans.  -Participants also reported differential treatment and bias in the faculty towards African Americans. |
| Facilitators and barriers to leadership and career opportunities in minority nurses in public health departments  Fowler BA | 2020 | USA | To examine minority public health nurses’ experiences and opportunities for leadership development and career advancement in public health departments. | n =39 minority nurses. | -Minority nurses reported experiences of both explicit and implicit racism in their workplace.  -Nurses also reported being negatively stereotyped as hostile and angry. |
| Getting In, Getting Hired, Getting Sideways Looks: Organizational Hierarchy and Perceptions of Racial Discrimination  Adia Harvey Wingfield & Koji Chavez | 2020 | USA | To examine the impact of racism in the workplace in occupational mobility. | n =60 interviews with various Black healthcare staff. | -Black workers’ status within an organizational hierarchy informed perceptions of the nature and type of workplace racial discrimination. Healthcare professionals higher up in the medical hierarchy focused mostly on organizational issue while healthcare professionals lower in the medical hierarchy focused more on experiences of racism in healthcare. |
| Patients, Pride, and Prejudice:  Exploring Black Ontarian Physicians' Experiences of Racism and Discrimination  Mpalirwa J, Lofters A, Nnorom O, Hanson MD | 2020 | Canada | To document Black physicians’ and physician trainees’ experiences of racism in healthcare in Ontario Canada. | n =46 online survey | -Race played an important role in career decisions especially in regards to speciality choice (presence of Black mentors in the given speciality).  -A majority of participants described negative experiences in regards to their race such as differential treatment in clinical settings compared to their White colleagues including having to work harder than others, being excluded, othered and being mistaken as cleaning staff.  -Participants reported lack of support in medical institutions in regards to their experiences of racism. As such participants had to get support from outside medical institutions. |
| ***Quantitative articles examining healthcare staff’s experiences of racism in healthcare (n=4)*** | | | | | |
| **Article title and author/s** | **Year of publication** | **Geographical location** | **Aims of articles** | **Methods** | **Key findings in regards to racism in healthcare** |
| African Americans in Oral and Maxillofacial Surgery: Factors Affecting Career Choice, Satisfaction, and Practice Patterns  Criddle, T.-R., Gordon, N. C., Blakey, G., & Bell, R. B | 2017 | USA | To explore factors that contribute to African American choosing Oral and Maxillofacial surgery as well as their satisfaction with their career choice. | n =41 survey answers (80 mailed surveys were sent. 41 surveys were included in the analysis). | -Most of the participants were satisfied with their residency training.  -A quarter of the informants indicated that they faced race related harassment in their workplace. However, the majority of the informants did not think race affected their residency applications. |
| Caregiver experiences of racism and child healthcare utilisation: cross-sectional analysis from New Zealand.  Paine, S.-J., Harris, R., Stanley, J., & Cormack, D. | 2018 | New Zealand | To examine the prevalence of racism in healthcare by child ethnicity; association between caregiver experiences of racism and child healthcare utilisation; and the contribution of caregiver socioeconomic position and psychological distress to this association. | n =4535 child–primary from 2006-2007 caregiver dyads. N=4420 dyads from 2011/2012. | -The prevalence of ‘any’ experience of racism was higher among caregivers of Maori and Asian children compared with those of European/Other children.  -Vicarious racism was independently associated with unmet need for child’s healthcare and dissatisfaction with their child’s medical centre. |
| Foreign-born physicians’ perceptions of discrimination and stress in Finland: a cross-sectional questionnaire study  Heponiemi, T., Hietapakka, L., Lehtoaro, S., & Aalto, A.-M | 2018 | Finland | To examine healthcare providers’ of foreign background experience. | n = 371 healthcare providers of foreign-background.  Discrimination was measured in terms of discrimination within the workplace from other staff members as well as patients. Other measurement included patient related stress, cross cultural training, cross cultural empathy, team climate, skill discretion and employment sector | -The majority of the informants were from East Europe.  -27% experienced discrimination often or sometimes from patients, while less discrimination was experienced from staff (10% from management and 12% from other co-workers).  - Analyses showed that respondents coming from Russia, other EU/EEA countries and other countries were more likely to experience discrimination from all sources than Respondents coming from Estonia.  -A better working climate was associated with less likelihood of discrimination from all sources. |
| Nativity Status and Workplace Discrimination in Registered Nurses: Testing the Mediating Role of Psychosocial Work Characteristics  Wesołowska, Karolina, Marko Elovainio, Kaisla Komulainen, Laura Hietapakka, and Tarja Heponiemi | 2020 | Finland | To explore whether foreign‐born and native nurses differed in frequency of workplace discrimination; and whether psychosocial work characteristics mediated the association between nativity status and the experience of workplace discrimination among registered female nurses. | n =610 registered female nurses of all ethnicities. | -Foreign‐born nurses reported higher frequency of workplace discrimination compared with native nurses.  -Foreign‐born nurses had lower levels of job control and lower levels of job demands than their native counterparts. |
| ***Mixed method articles examining healthcare staff’s experiences of racism in healthcare (n=1)*** | | | | | |
| **Article title and author/s** | **Year of publication** | **Geographical location** | **Aim of article** | **Methodology** | **Key findings in regards to racism in healthcare** |
| Racism as Experienced by Physicians of Color in the Health Care Setting  Serafini K, Coyer C, Brown Speights J, Donovan D, Guh J, Washington J, Ainsworth C | 2020 | USA | To examine the impact of racism in the workplace among physicians of color in the USA. | *Qualitative*  n =71 physicians.  *Quantitative*  n =71 physicians. | -23.3% pf physicians reported experiencing patient refusal due to their ethnicity  -21.9% of physicians reported experiencing patient refusal but were unsure if it was due to their ethnicity.  -Microaggressions were positively correlated with a measure of secondary traumatic stress, racism from patients.  -Participants who reported English as their second language reported significantly more instances of racism from patients  than those who spoke English as a first language.  -Qualitative data showed that physicians reported microagressions, being held to higher standards, institutional neglect, differential treatment and negative assumptions on their educational level. |
| ***Qualitative articles examining healthcare staff racial attitudes and beliefs (n=12)*** | | | | | |
| **Article title and author/s** | **Year of publication** | **Geographical location** | **Aims of articles** | **Methods** | **Key findings in regards to racism in healthcare** |
| Meeting the needs of minority ethnic patients.  Cortis, Joseph D. | 2004 | UK | How healthcare providers view healthcare users from ethnic minorities (Pakistani patients). | n = 30 nurses. | -Challenges were described in healthcare when meeting Pakistani patients such as difficulty in understanding the Pakistani community and poor preparation to meet the needs of ethnic diverse society and presence of racism in practice setting. |
| Nurses’ experiences of caring for South Asian minority ethnic patients in a general hospital in England  Vydelingum, V | 2006 | UK | To explore how healthcare providers view South Asian minority ethnic healthcare users. | n = 43 interviews (22 nurses and 18 care assistants). | -A tendency to homogenise minority ethnic patients and the put the blame on the ethnic minorities in healthcare encounters while simultaneously deny the existence of racism.  -A tendency to view other religious beliefs (Islam, Hinduism and Judaism) as irrational.  -Participants referred to racism as bigotry. |
| Cultural diversity in the Dublin maternity services: the experiences of maternity service providers when caring for ethnic minority women  Lyons, S. M., O’Keeffe, F. M., Clarke, A. T., & Staines, A. | 2008 | Ireland | To explore the experiences of Irish maternity service providers when caring for ethnic minority women. | n =15 maternity healthcare providers. | -The main issue identified by healthcare providers when communicating with ethnic minority was language difficulties.  -Providers discussed that ethnic minority women did not conform to “unwritten” hospital rules and were perceived as demanding.  -Providers also stated that ethnic minority women had different ways of dealing with labour and that they were too dramatic. However, providers praised these women for their breastfeeding habits.  -Providers perceived ethnic minority women as different from “them” and reported having negative emotive feeling such as stress and worry when dealing with ethnic minority women.  -Providers did not see their perceptions of ethnic minority patients as racism and stated that although ethnic minority women were rude and difficult; these feelings were real and thus cannot be attributed to racism.  -In general, ethnic minority women were expected to adapt to the existing healthcare system instead of the healthcare system being responsive to patients’ needs. |
| Health Providers’ Narratives on Intimate Partner Violence Against Roma Women in Spain  Briones-Vozmediano, E., La Parra-Casado, D., & Vives-Cases, C | 2018 | Spain | To explore healthcare professionals perceptions of Roma people in healthcare focusing on Roma people facing intimate partner violence. | n = 25 with healthcare professionals | -Roma people were characterised as the other culture and providers viewed Roma people as marginalised and suffering from social exclusion. However, most providers had negative perceptions about Roma people and viewed them as trying to use healthcare for the sake of benefits.  -Intimate partner violence among Roma people was seen as due to the more patriarchal nature of Roma and thus intimate partner violence among Roma people was normalised by healthcare providers.  -Providers demonstrated discriminatory perceptions about Roma women such as stating that Roma women do not receive healthcare because they do not want it. |
| Student nurse perceptions of Gypsy Roma Travellers; A European qualitative study  Heaslip V, Vanceulebroeck V, Kalkan I, Kömürcü N, Solanas IA | 2019 | UK, Spain, Belgium and Turkey | To explore student nurses’ perceptions on Roma travellers. | n = 23 student nurses from 4 Europeans countries. | -Most of the students did not have professional contact with Roma travellers. However, they still articulated negative views towards these communities. |
| Inequality and discrimination in access to urgent care in France  Ethnographies of three healthcare structures and their audiences  Morel S | 2019 | France | To examine how the organization of emergency care in France contributes to inequalities and discrimination in access to care. | *Qualitative*  Ethnographic observations (2005-2011) in a private [ambulance](https://www-sciencedirect-com.ezproxy.its.uu.se/topics/medicine-and-dentistry/ambulance) company, a public emergency department, and a private, for-profit emergency department. Interviews with healthcare professionals and immigrants (2017)  n = 16 healthcare professionals  n =14 immigrants.  *Quantitative*  2007 survey with patients.  n =451 patients from both private and public sector. | -The private and public emergency departments avoid certain patient profiles.  -Patients who are deemed undesirable are put in a different socio-medical assistance pathway.  -The emergency healthcare system reproduces social inequalities. |
| Experiences of Community Doulas Working with Low-Income, African American Mothers  Wint K, Elias TI, Mendez G, Mendez DD, Gary-Webb TL | 2019 | USA | To explore doulas’ experiences with interacting with low-income African American women. | n =10 Doulas. | -Doulas discussed that similarities of race and cultural experience aids in establishing a trustful relationship between doulas and African American women.  -Doulas also talked about helping patients in navigating Institutional biases within the health care system. |
| Confronting racism in family planning: A critical ethnography of Roma health mediation  Kühlbrandt, C | 2020 | Romania | To examine the Roma health mediation programme which focuses on combating discrimination against Roma. | Ethnographic observations + n =40 interviews with various healthcare staff. | -Healthcare professionals had negative views about Roma women. These views included a perception that Romani women were forced to conceive and that they did not care about contraception as well as a perception that Romani women had children for the sake of getting child benefits.  -The perceptions shared by healthcare professionals did not resonate with Roma women. Roma women reported struggling with accessing contraceptives due to financial constraints. |
| Why don't health care frontline professionals do more for  segregated Roma? Exploring mechanisms supporting unequal care practices  Belak A, Filakovska Bobakova D, Madarasova Geckova A, van Dijk JP, Reijneveld SA. | 2020 | Slovakia | To explore what kinds of substandard practices health care frontline professionals engage in regarding  segregated Roma –To explore what mechanisms of support practices healthcare professionals use in caring for Roma people. | n = 23 healthcare professionals serving Roma people. | -Negative attitudes towards Roma people were identified as one of the mechanisms of substandard care. These attitudes included stereotypes about Roma people being demanding, weird and immutable people.  -Overt racism was also identified including invasive, laxer and offensive treatment of Roma patients |
| Perspectives and Experiences of Obstetricians Who Provide Labor and Delivery Care for Micronesian Women in Hawai'i: What Is Driving Cesarean Delivery Rates?  Delafield R, Elia J, Chang A, Kaneshiro B, Sentell T, Pirkle CM. | 2020 | USA | To describe obstetrician–  gynecologists’ perspectives on labor and delivery care for Micronesian women in Hawai‘i and possible. | n =13 interviews with obstetrician–  gynaecologists. | -Participants discussed how stereotypes and racial prejudice played a role in healthcare communications with Micronesian women.  -Participants discussed stereotypes about Micronesian women having more infectious diseases and being dirty.  -Participants discussed hearing derogatory terms about Micronesian women.  -Participants also reflected upon their racial bias towards Micronesian women. |
| Culturally Sensitive Care: Definitions, Perceptions, and Practices of Health Care  Professionals  Claeys A, Berdai-Chaouni S, Tricas-Sauras S, De Donder L | 2020 | Belgium | To explore healthcare professionals perceptions of cultural sensitive care in Belgium. | n =34.  Focus group discussion with healthcare professionals. | -Participants perceived cultural sensitive care narrowly and focused on specific groups of migrants when speaking about cultural sensitive care i.e., the Islamic religion and/or Arabic culture.  -Participants othered certain migrant groups (micro-racism) and showed little empathy towards these groups.  -Participants perceived a lack of competence to oppose racism toward patients from their colleagues. |
| ‘With my heart and eyes open’: Nursing students′ reflections on placements in Australian, urban aboriginal organisations  Tamara Power, Cherie Lucas, Carolyn Hayes, Debra Jackson | 2020 | Australia | To explore the experiences of nursing students in Australia with working with indigenous people. | n =8 nursing students. | -Nursing students described how their perceptions about indigenous people were shaped by negative media and community stereotypes.  -Nursing students also described feeling apprehensive about working with indigenous people. |
| ***Quantitative articles examining healthcare staff racial attitudes and beliefs (n=20)*** | | | | | |
| **Article title and author/s** | **Year of publication** | **Geographical location** | **Aims of articles** | **Methods** | **Key findings in regards to racism in healthcare** |
| Physicians’ implicit and explicit attitudes about race by MD race, ethnicity, and gender  Sabin, J., Nosek, B. A., Greenwald, A., & Rivara, F. P. | 2009 | USA | To examine physicians' implicit racial attitudes. | n =2,535 medical doctors. | -Medical doctors demonstrated an implicit preference for White Americans relative to Black Americans.  - Strength of implicit bias exceeded self-report among all test takers except African American medical doctors who did not show an implicit preference for either Blacks or Whites.  -Women showed less implicit bias than men. |
| Racial-ethnic biases, time pressure, and medical decisions.  Stepanikova, I | 2012 | USA | -To examine implicit bias and time pressure and its effect on implicit bias. | n = 81 family and general internists physicians.  -Case vignette was used where a patient with chest pain was described. Time pressure was manipulated experimentally. | -Implicit bias led to less serious diagnosis in regards to Black and Hispanics when time pressure was high but not when it was low.  - Under high pressure, implicit bias in regards to Black patients was associated with a lower likelihood of a referral to a specialist. |
| Clinicians’ implicit ethnic/racial bias and perceptions of care among Black and Latino patients  Irene V. Blair, John F. Steiner, Diane L. Fairclough, Rebecca Hanratty, David W. Price, Holen K. Hirsh, Leslie A. Wright, Michael Bronsert, Elhum Karimkhani, David J. Magid and Edward P. Havranek | 2013 | USA | To examine whether clinicians’ explicit and implicit ethnic/racial bias is related to Black and Latino patients’ perceptions of their healthcare in clinical relationships. | n =2,908 patients and  n =134 clinicians. Clinicians were tested for both implicit and explicit racial bias. | -Clinicians had low levels of explicit bias. Explicit bias was not related to patients’ perceptions.  -Implicit bias varied among clinicians.  -Clinicians with greater implicit bias rated lower in patient-entered care by their Black patients as compared with a reference group of White patients (Black patients rated clinicians who scored 1.0 on the implicit association test approximately 6 points lower on interpersonal treatment than clinicians who scored 0 on the test). |
| Unconscious race and class bias: its association with decision making by trauma and acute care surgeons  Haider, A. H., Schneider, E. B., Sriram, N., Dossick, D. S., Scott, V. K., Swoboda, S. M., … Cooper, L. A. | 2014 | USA | To determine whether race and class bias exists among trauma/acute surgeons  -To determine whether this bias affects surgeons’ clinical decisions. | A prospective Web-based survey n =248 members of the Eastern Association for the Surgery of Trauma  -9 clinical vignettes were completed by informants. Association Test (IAT) and social class IAT assessments were completed by each participant. | - 79% surgeons explicitly stated that they had no race preferences and 55% stated that they had no social class preference.  - 73.5% of the participants had IAT scored reporting implicit bias towards White patients and 90.7% upper class patients.  - Multivariable analyses revealed no relationship between IAT scores and vignette-based clinical assessments. |
| Cultural competency, race, and skin tone bias among pharmacy, nursing, and medical students: implications for addressing health disparities  White-Means, S., Zhiyong Dong, null, Hufstader, M., & Brown, L. T. | 2014 | USA | To measure, compare, and contrast objective and subjective cognitive processes among pharmacy, nursing, and medical students to discern  potential implications for health disparities. | n = 189 pharmacy students. N=29 1^st^ year nursing students. n = 115 medical students  Cultural competency questionnaire and two implicit racial bias tests were used. | -Results showed that there was significant racial implicit bias in favour of Whites and light skinned patients.  -62% of non-Hispanic blacks (African Americans and Africans) had a preference for Whites compared to blacks.  - 94% of non-Hispanic Whites, 100% of Hispanics, and 76% of Asians had a preference for Whites over Blacks. |
| Do physicians’ implicit views of African Americans affect clinical decision making?  Oliver, M. N., Wells, K. M., Joy-Gaba, J. A., Hawkins, C. B., & Nosek, B. A | 2014 | USA | - To determine whether physicians’ implicit influences decision concerning total knee transplant among African Americans. | n = 543 family and internal medicine physicians  The physicians were given a scenario describing either a Black or White patient with severe osteoarthritis refractory to medical treatment. | -There was strong implicit bias towards White patients in comparison to blacks.  -Physicians reported Whites to be more cooperative than blacks.  -Implicit bias did not predict choice of treatment in regards to knee transplants. |
| Unconscious Race and Class Biases among Registered Nurses: Vignette-Based Study Using Implicit Association Testing  Adil H Haider, Eric B Schneider, N Sriram, Valerie K Scott, Sandra M Swoboda, Cheryl K Zogg, Nitasha Dhiman, Elliott R Haut, David T Efron , Peter J Pronovost, Julie A Freischlag, Pamela A Lipsett, Edward E Cornwell, Ellen J MacKenzie, Lisa A Cooper | 2015 | USA | To determine whether nurses have implicit racial and class bias. | n =245 Nurses  -Implicit associate tests was used to analyse data.  -Clinical vignettes were used to determine whether implicit racial and class bias influenced clinical decisions. | -The majority of nurses reported that they had no racial or class bias.  -After applying implicit association tests, the majority of nurses showed both racial (85.3%) and class (93.47%) bias.  -Implicit association tests scores did not statistically correlate with vignette-based clinical decision-making. |
| Examining implicit bias of physicians who care for individuals with spinal cord injury: A pilot study and future directions  Leslie R M Hausmann, Larissa Myaskovsky, Christian Niyonkuru, Michelle L Oyster, Galen E Switzer, Kelly H Burkitt, Michael J Fine, Shasha Gao, Michael L Boninger | 2015 | USA | To examine whether spinal cord injury healthcare providers exhibit implicit bias towards patients. | n =162 patients with spinal cord injury  n =14 physicians  Implicit Association Test (IAT) was used to assess physicians’ implicit bias  Participants with spinal cord injury were matched with physician whom they saw most frequently during the study timeframe. | -The majority of patients with spinal cord injury were White (77%).  -All physicians implicitly associated positive concepts with being White compared to being black.  -Physician implicit racial bias was associated with greater likelihood of disability in social integration and higher levels of depression and lower life satisfaction. |
| The Impact of Cognitive Stressors in the Emergency Department on Physician Implicit Racial Bias  Tiffani J Johnson, Robert W Hickey , Galen E Switzer, Elizabeth Miller, Daniel G Winger, Margaret Nguyen, Richard A Saladino, Leslie R M Hausmann | 2016 | USA | To determine the impact stress has on racial bias among physicians in emergency departments. | n =91 physicians in paediatric emergency department.  -Implicit association test was used. | -Participants displayed moderate pro-White/anti-Black bias on pre-shift and post-shift IAT scores.  -Implicit association bias scores did not change pre and post shift. However, implicit racial bias increased when the shift was overcrowded (caring for more than 10 patients). |
| The Role of Bias by Emergency Department Providers in Care for American Indian Children  Puumala, S. E., Burgess, K. M., Kharbanda, A. B., Zook, H. G., Castille, D. M., Pickner, W. J., & Payne, N. R. | 2016 | USA | -To assess both explicit and implicit bias towards American Indian children. | n = 154 care providers  -Explicit bias was assessed with questions included  American Indian stereotypes.  -Implicit Association Test was used.  -Clinical vignettes were used to assess clinical decisions. | -84% of the providers had an implicit preference for non-Hispanic White adults or children.  -American Indian children were seen as increasingly challenging and parents/caregivers less compliant. |
| Is Allison More Likely Than Lakisha to Receive a Callback From Counseling Professionals? A Racism Audit Study  Shin, R. Q., Smith, L. C., Welch, J. C., & Ezeofor, I. | 2016 | USA | -To examine racially biased call-back responses in in mental health. | n = 371 calls  Actor were used to represent patients presenting themselves with names representing two races. Allison to represent a White non-Latino patient and Lakisha to represent a Black non-Latino patient. | -198 were placed for Allison and 173 were placed for Lakisha.  -Allison and Lakisha received 131 and 99 call-backs so no statistical differences was observed in call-backs.  -The caller with the stereotypically White-sounding name received voice messages that promoted the potential for services at a 12% higher rate than the caller with the stereotypically Black-sounding name. |
| Physician Racial Bias and Word Use during Racially Discordant Medical Interactions.  Hagiwara, N., Slatcher, R. B., Eggly, S., & Penner, L. A. | 2017 | USA | To examine physician’s racial bias relation with word use during medical interaction with Black patients | n=117 video-recorded medical interactions. Computer-based Race Implicit Association Test was used to analyse the data. Explicit bias was also assessed at baseline. Linguistic Inquiry and Word Count was utilized to compute the percentage of words in two broad categories of word types used by the physician in each interaction: first-person pronouns and emotion-related words. | -Physicians with higher levels of implicit racial bias used first-person plural pronouns and anxiety-related words more frequently than physicians with lower levels of implicit bias.  -Physicians with higher levels of explicit racial bias tend to use first-person singular pronouns more frequently than physicians with lower levels of explicit bias.  -Findings suggest that non-Black physicians with higher levels of implicit racial bias may tend to use more words that reflect social dominance (first-person plural pronouns) and anxiety when interacting with Black patients. |
| Informal Training Experiences and Explicit Bias against African Americans among Medical Students  Burke, S. E., Dovidio, J. F., Perry, S. P., Burgess, D. J., Hardeman, R. R., Phelan, S. M., … van Ryn, M. | 2014 | USA | To explore the attitudes of White medical students towards African Americans in the beginning and end of their education. | n = 2,922 Non-African American medical students. | -Students mostly evaluated African Americans as favourable. However, they evaluated African Americans as less favourable than Whites.  -Contact with African Americans was an important predictor for positive attitudes towards African American. |
| Comparison of Physician Implicit Racial Bias Toward Adults Versus Children  Tiffani J Johnson, Daniel G Winger, Robert W Hickey, Galen E Switzer, Elizabeth Miller, Margaret B Nguyen, Richard A Saladino, Leslie R M Hausmann | 2017 | USA | -To compare physician’s implicit bias towards black’s adults with implicit bias towards Black children. | n =91 physicians  Implicit association bias test was used. | -Moderate pro-White/anti-Black bias on both the Adult D=0.34) and Child Race implicit association bias test.  -There was no significant difference between Adult and Child Race implicit association bias scores.  -Implicit bias was not associated with resident demographic characteristics, including specialty. |
| Ethnic bias and clinical decision-making among New Zealand medical students: an observational study  Ricci Harris, Donna Cormack, James Stanley, Elana Curtis, Rhys Jones, Cameron Lacey | 2018 | New Zealand | To examine ethnic bias among medical students in New Zealand | n =302 medical students  Implicit bias test was conducted.  Clinical vignettes were used and patients’ ethnic group was manipulated. | -Medical students demonstrated both an explicit and implicit ethnic bias in favour of New Zealand Europeans.  -In the clinical vignettes no significant differences in clinical decision-making by patient ethnicity were observed. |
| Ethnic bias amongst medical students in Aotearoa/New Zealand: Findings from the Bias and Decision Making in Medicine (BDMM) study  Cormack, D., Harris, R., Stanley, J., Lacey, C., Jones, R., & Curtis, E. | 2018 | New Zealand | To examine medical students’ racial bias. | n =302 medical students. Two chronic disease vignettes, two implicit bias measures, and measures of explicit bias were used. | -Participants demonstrated a pro-European New Zealand racial bias and bias on viewing Europeans as more compliant than Maori people.  -Explicit bias was less common. |
| A comparison of clinicians’ racial biases in the United States and France  Khosla, N. N., Perry, S. P., Moss-Racusin, C. A., Burke, S. E., & Dovidio, J. F. | 2018 | Multicounty (USA and France) | -To examine healthcare providers’ perceptions of Black versus White patients’ personal responsibility for their health. | n =164.  n =81 French clinicians  n =83 American clinicians. | -French clinicians did not exhibit significant racial bias on the measures if interests.  -American clinicians rated a hypothetical White patient to more likely improve health wise compared to Black patients.  -In the USA personal responsibility mediated the racial difference in expected improvement. A White person was viewed as being more personally responsible than Black patients. |
| Incoming Medical Students' Political Orientation Affects Outcomes Related to Care of Marginalized Groups: Results from the Medical Student CHANGES Study  Burgess, D. J., Hardeman, R. R., Burke, S. E., Cunningham, B. A., Dovidio, J. F., Nelson, D. B., Ryn, M., Perry, Sylvia, Phelan, Sean M, Yeazel, Mark W, Herrin, Jeph, van Ryn, Michelle | 2019 | USA | To examine whether political orientation is related to implicit bias against marginalised groups. | n =3756 first and four year students in 2010 and 2011. | - More conservative ideology was associated at year 4 with greater implicit bias against Black and gay individuals, more negative explicit attitudes toward stigmatized groups, lower internal motivation to control racial prejudice, lower levels of trait empathy and empathy toward patients, and lower levels of patient-centered attitudes. |
| Are symptoms of burnout associated with resident physicians’ implicit and explicit biases toward black people?  Dyrbye L, Herrin J, West CP, Wittlin NM, Dovidio JF, Hardeman R, Burke SE,  Phelan S, Onyeador IN, Cunningham B, van Ryn M | 2019 | USA | To determine is symptoms of burnout are associated with resident physicians’ implicit and explicit biases toward Black people. | n =3392 second-year resident physicians who self-identified as non-Black and who have had symptoms of burnout. | -Findings show that burnout time point was associated with greater explicit and implicit racial biases.  -After adjusting for demographics, specialty, depression, and feeling thermometer scores toward White people, resident physicians with burnout had greater explicit racial bias.  -Higher emotional exhaustion and depersonalization scores were associated with more unfavorable attitudes toward Black people. |
| Racial Disparity in the Clinical Risk Assessment  Kerner, J., McCoy, B., Nadia, G., Colavita, M., Kim, M., Zaval, L., & Merrill, R | 2020 | USA | To examine psychiatrics’ implicit bias in the emergency room through examining admission rates of various ethnic groups. | n =743 psychiatrics. | -No difference was found in emergency admission rate among various ethnic/racial groups. |
| ***Quantitative articles examining effects of racism in healthcare on various treatment choices (n=13)*** | | | | | |
| **Article title and author/s** | **Year of publication** | **Geographical location** | **Aim of study** | **Methods** | **Key findings in regards to racism in healthcare** |
| The effect of cognitive load and patient race on physicians’ decisions to prescribe opioids for chronic low back pain: a randomized trial  Burgess, Diana J., Phelan, S., Workman, M., Hagel, E., Nelson, D. B., Fu, S. S., … van Ryn, M. | 2014 | USA | -To examine whether work load affects healthcare providers’ implicit bias. | n =98 healthcare providers  Web-based experimental study with randomly assigned case vignettes where the patients race was changed (Black/White). | **-**Gender played a role in opioid prescription where male physicians were less likely to prescribe opioids for Black than White patients under high cognitive load and were more likely to prescribe opioids for Black than White patients under low cognitive load.  -Female physicians were more likely to prescribe opioids for Black than White patients in both conditions, with greater racial differences under high vs low cognitive load. |
| The impact of patient race on clinical decisions related to prescribing HIV pre-exposure prophyla1xis (PrEP): assumptions about sexual risk compensation and implications for access  Calabrese, S. K., Earnshaw, V. A., Underhill, K., Hansen, N. B., & Dovidio, J. F | 2014 | USA | To explore racial bias effect on clinical decision regarding prescription of HIV exposure prophylaxis. | n = 102 students  Clinical Vignettes were used and patients’ race was manipulated. | -Students judged Black patients as more likely to engage in HIV pre-exposure prophylaxis associated sexual risk behaviour in comparison to White patients.  -The previous judgment was associated with less likelihood of prescribing treatment. |
| Racial bias in pain assessment and treatment recommendations, and false beliefs about biological differences between blacks and whites  Hoffman, K. M., Trawalter, S., A1t, J. R., & Oliver, M. N. | 2016 | USA | To examine whether pain assessment is related to a perception of biological differences between Black and White. | Study 1:  Patients: n =121  Participants were asked to rate their pain experience in various scenarios. Participants were also asked to rate whether biological differences between Black and Whites are true or untrue.  Providers.  n = 418 medical students and residents. | -*Study 1*: This study showed that White healthcare users endorsed some beliefs about biological differences between blacks and Whites.  -*Study 2*: This study showed that many White medical students and residents hold beliefs about biological differences between blacks and Whites, many of which are false and that these false beliefs are in fact related to racial bias in pain perception. |
| The Effects of Oncologist Implicit Racial Bias in Racially Discordant Oncology Interactions  Louis A Penner, John F Dovidio, Richard Gonzalez, Terrance L Albrecht, Robert Chapman, Tanina Foster, Felicity W K Harper, Nao Hagiwara, Lauren M Hamel, Anthony F Shields, Shirish Gadgeel, Michael S Simon, Jennifer J Griggs, Susan Eggly | 2016 | USA | To examine whether racial bias affects interaction between patients and providers in oncology. | n = 112 Black patients  n = 18 non-Black medical oncologists.  Implicit bias test was completed by providers. Oncologist communication and interaction length of time with patients was also recorded. | -Oncologists who measured high in implicit racial bias had shorter interactions with Black patients.  -Higher implicit racial was also associated with more patient difficulty in remembering contents of interaction.  -Implicit bias also indirectly predicted patients’ confidence in recommending treatment and difficulties in completing them. |
| The effects of racial attitudes on affect and engagement in racially discordant medical interactions between non-Black physicians and Black patients  Hagiwara, N., Dovidio, J. F., Eggly, S., & Penner, L. A | 2016 | USA | To analyse recorded medical interactions between Black patients and non-Black providers. | n =113 interactions. | -Physicians’ affect and engagement were influenced by their implicit and explicit racial bias but only when they interacted with patients who reported any incidence of prior discrimination.  -Patients’ affect was only influenced by perceived discrimination. |
| Obstetrical Providers’ Management of Chronic Pain in Pregnancy: A Vignette Study.  Tucker Edmonds, B., McKenzie, F., Austgen, M. B., Ashburn-Nardo, L., Matthias, M. S., & Hirsh, A. T | 2017 | USA | -To describe providers’ management of a hypothetical case on chronic pain in pregnancy to determine which practices differ by race. | n =76  A case-vignette of a woman suffering from chronic pain during pregnancy. “Race” was altered (Black and White) and providers’ responses in terms of opioid prescription was recorded. | -Providers were more likely to conduct urine tests on White patients in comparison with Black patients.  -Providers were more likely to suspect that White patients would divert the medication.  -Providers’ highest concern with White patients was their risk of abuse or addiction.  -Providers’ highest concern with Black patients was harm to the fetus.  -Suspicion about symptom exaggeration was more closely related to decisions about refilling the opioid prescriptions and increasing the dose for Black patients, whereas these decisions were more closely correlated with concerns about overdose for White patients. |
| "Are you accepting new patients?" A pilot field experiment on telephone-based gatekeeping and Black patients' access to pediatric care  Leech TGJ, Irby-Shasanmi A, Mitchell AL | 2019 | USA | To determine whether name and accent cues that the caller is Black shape physician offices' responses to telephone-based requests for well-child visits. | n =205 audits. | -Black auditors were less likely to be told an office was accepting new patients and were more likely to experience both withholding behaviors and misattributions about public insurance in comparison to White auditors. |
| Racial and insurance-related disparities in delivery of immunotherapy-type compounds in the united states  Verma, V., Haque, W., Cushman, T. R., Lin, C., Simone, C. B., Chang, J. Y., . . . Welsh, J. W | 2019 | USA | To examine factors associated with inequalities in the receipts of immunotherapy type compounds. | n = 504,447 patients who were newly diagnosed with cancer. | -Analysis showed racial inequalities regarding treatment with immunotherapy compounds.  -African American were less likely to receive treatment with immunotherapy compounds independent of insurance. |
| Identification of Racial Inequities in Access to  Specialized Inpatient Heart Failure Care at an Academic Medical Center  Eberly LA et al. | 2019 | USA | To examine the relationship between patients’ race and admission rates in cardiology. | n =1967 (66.7% White, 23.6% black, and 9.7% Latinx). | -Black and Latinx patients had lower rates of admission to the cardiology service compared to White patients.  -Female sex and age >75 years were also independently associated with lower rates of admission to the cardiology service. |
| Racial/ethnic disparities in atrial fibrillation treatment and outcomes among dialysis patients in the united states  Salina P. Waddy, Allen J. Solomon, Adan Z. Becerra, Julia B. Ward, Kevin E. Chan, Chyng-Wen Fwu, Jenna M. Norton, Paul W. Eggers, Kevin C. Abbott and Paul L. Kimmel | 2020 | USA | To investigate racial inequalities in stroke and End Stage Kidney disease treatment. | n = 56,587 End Stage Kidney Disease hemodialysis patients with atrial fibrillation 2006 to 2013. | -Black, Hispanic and Asian patients were more likely to experience stroke compared to White patients but less likely to fill a warfarin prescription. |
| Racial Disparities in End-of-Life Care Between Black and White Adults With Metastatic Cancer  Perry LM, Walsh LE, Horswell R, Miele L, Chu S, Melancon B, Lefante J, Blais CM, Rogers JL, Hoerger M | 2020 | USA | To examine whether Black adults received more burdensome end of life care than White adults in a population-based  data set of cancer decedents in Louisiana USA. | n =875 White and 415 Black patients. | - Odds ratios indicated that Black patients were more likely than White individuals to be hospitalized or admitted to the emergency department during the last month of life.  -Black patients received more burdensome care than White patients. |
| Racial and ethnic disparities in severe maternal morbidity and anesthetic techniques for obstetric deliveries: A multi-state analysis, 2007–2014  Tangel, V. E., Matthews, K. C., Abramovitz, S. E., & White, R. S. | 2020 | USA | To evaluate racial and ethnic disparities in severe maternal morbidity and administered  anesthesia techniques. | n = 6,879,332 patients aged ≥18 years old who underwent deliveries were identified by International  Classification of Disease, 9th Revision between 2007 and 2014. | -Black women were more likely than White women to experience severe maternal morbidity when controlling for patient demographics, comorbidities, and hospital characteristics. This finding was consistent in stratified analyses.  -Black women were more likely than White women to receive general anesthesia for cesarean delivery and to receive no analgesia for vaginal delivery. |
| Physician-patient racial concordance and disparities in birthing mortality for newborns  Greenwood, B. N., Hardeman, R. R., Huang, L., & Sojourner, A | 2020 | USA | To examine the association between physician-patient concordance and new born mortality rate. | n =1.8 million hospital births in Florida between 1992 and 2015. | - Newborn–physician racial concordance is associated with a significant improvement in mortality for Black infants.  -These positive effects of physician newborn racial concordance manifest strongly in more complicated cases and when hospitals deliver more Black newborn. |
| ***Mixed method articles examining effects of racism in healthcare on various treatment choices (n=1)*** | | | | | |
| Does Race Influence Decision Making for Advanced Heart Failure Therapies?  Khadijah Breathett; Erika Yee, Natalie; Megan Hebdon; Janice D. Crist; Shannon Knapp; Ashley Larsen; Sade Solola,; Luis Luy; Kathryn Herrera-Theut; Leanne Zabala,; Jeff Stone; Marylyn M. McEwen; Elizabeth Calhoun; Nancy K. Sweitzer | 2019 | USA | To determine whether race is associated with heart failure treatment. | *Quantitative*  n =422 participants in 2018  *Qualitative*  n =42 participants | -The survey showed no difference in racial rating for advanced therapies.  -Interviews showed that race influenced all steps of the decision making process. |
| ***Qualitative articles examining healthcare staffs’ reflections on racism in healthcare (n=15)*** | | | | | |
| **Article title and author/s** | **Year of publication** | **Geographical location** | **Aims of articles** | **Methods** | **Key findings in regards to racism in healthcare** |
| Is it racism? Skepticism and resistance towards ethnic minority care workers among older care recipients  Jönson, H | 2007 | Sweden | To explore how healthcare professionals view racism. | n =12 care givers were interviewed | -Racism was seen as a sensitive issue by the participants.  -Racism was viewed as rare but does exist as described by the majority group who framed racism as mostly about fear of the unknown. In contrast care givers with a minority background described the issue as frequent.  -Ethnic minority care givers endured care recipients negative complaints about migrants. These negative view included viewing migrants as exploiting the welfare system, committing crimes, taking jobs from Swedes, being unclean, ungrateful and should be sent back to where they came from.  -Issues with racism were reported to disappear once the care giver and client got to know each other.  -Complaints about language in regards to ethnic minority’s language abilities was also reported by managers.  -All representatives embraced the official norm of anti-racism. |
| Service providers' perspectives, attitudes and beliefs on health services delivery for Aboriginal people receiving haemodialysis in rural Australia: a qualitative study  Rix, Elizabeth F. Barclay, Lesley Wilson, Shawn Stirling, Janelle Tong, Allison | 2013 | Australia | To examine how healthcare professionals view racism in regards to Haemodialysis Aboriginal patients. | n =29 healthcare providers (mostly non- Aboriginal but also Aboriginal). | - Some participants believed that individual racism was a problem, while others considered racism to be part of the Australian ‘White’ culture, defining it as historical suspicion of a person of another colour or cultural background.  -Most agreed that racism was a barrier to effective communication and their ability to develop positive relationships with Aboriginal patients.  -A few participants mentioned that Aboriginal people could recognise ‘racist’ body language, which contributed to patients’ distress and insecurities.  -Participants stated that a system which does not accommodate the need of aboriginal people is a racist system. |
| Race matters: perceptions of race and racism in a sickle cell center.  Nelson, S. C., & Hackman, H. W | 2013 | USA | To explore both patients and providers perceptions of race and racism focusing on sickle cell disease. | n =112 Patients  n = 135 Healthcare providers. | -The majority of patients identified as Black and the majority of providers identified as White.  -More patients perceived that racism influenced healthcare quality.  -More staff perceived unequal treatment within healthcare settings.  -Although staff perceived racism as a problem in the USA, they did not perceive it as a problem within their institution. |
| Intersectional perspective in elderly care  Marta Cuesta | 2016 | Sweden | To explore how intersectionality may help healthcare professionals in their work. | n =10 interviewees with 8 assistant nurse and 2 nurses. | Three themes emerged from the data: 1) Intersectionality, knowledge, and experiences of professionalism; 2) Intersectionality, knowledge, and experiences of collaboration; and 3) Intersectionality, knowledge, and experiences of discrimination.  -Intersectional perspectives were perceived to make visible experiences of discrimination and develop better understanding of the issue. |
| Neutrality in medicine and health professionals from ethnic minority groups: The case of Arab health professionals in Israel  Keshet, Y., & Popper-Giveon, A | 2017 | Israel | To examine healthcare providers experiences of racism within healthcare settings. | n =33 interviews with Arab healthcare professionals. | -Racism from patients was reported.  -Medicine was perceived as rational and neutral and based on natural science. Additionally, there was a desire to care for people and treat them. All patients were treated the same regardless of their ethnicity and religion. However, some patients refused to be treated by Arab healthcare professionals.  -Difficulty in getting promoted and developing professionally especially for Arab professionals was reported as well as feelings of discrimination when working in mixed teams i.e., Arabs and Israeli together. |
| Mixed and misunderstandings: An exploration of the meaning of racism with maternal, child, and family health nurses in South Australia  Grant, J., & Guerin, P. B | 2018 | Australia | To explore how healthcare professionals view racism | n =31 Maternal child and family health nurses. | –Participants lacked clarity in defining racism. Racism was understood to mean discrimination based on race, religion and color. In some cases, it was conflated with gender, age and poverty. Racism was seen as about judgments based on assumptions and preconceptions as well as irrational emotional response as anger and hate.  -There were mixed views on whether infants and toddlers could experience racism or notice differences. Some stated that children could not experience racism at an early age while others thought that children were good at body language and could be affected by negative views.  -Participants questioned whether the healthcare service they provided was racist and had an impact on choice and relationship building. |
| Community nurses’ talk of equality and the discursive constitution of selves  Aranda, K | 2018 | UK | To explore how community nurses talked about equal opportunities, antidiscrimination in healthcare. | Qualitative interviews:  n =14 nurses and 14 nursing students. | -Participants drew on notions of solidarity and equality of healthcare. Nurses constructed themselves as neutral and impartial despite resources’ issues. However, nurses talked about the danger of having prejudice and discussed the importance of self-reflection. |
| Colonial legacies and collaborative action: Improving indigenous peoples' health care in Canada  Wylie, L., McConkey, S., & Corrado, A. M | 2019 | Canada | To examine the barriers that indigenous people in Canada face when accessing healthcare. | n =31 participants including 8 indigenous people and 22 non-indigenous healthcare professionals. | - Many participants discussed how social determinants of health, such as poverty, rural isolation, physical environments, and racism as having an adverse impact on Indigenous people’s health, and as creating barriers to accessing healthcare.  -Participants reported negative stereotypes by healthcare providers such as blaming indigenous people for their health status. These negative stereotypes contributed to distrusting the healthcare system. |
| The tools at their fingertips: How settler colonial geographies shape medical educators' strategies for grappling with Anti-Indigenous racism  Sylvestre P, Castleden H, Denis J, Martin D, Bombay A | 2019 | Canada | To explore how medical educators perceive issues of race and racism in regards to indigenous people. | n = 14 educators (8 professional competency training educators and 5 educators who direct clerkships). | -Participants who spent time working with indigenous people showed a more nuanced understanding of indigenous people’s realities.  -Participants who did not work with indigenous people tended to reproduce negative stereotypes in regards to indigenous people and their health issues. |
| Insiders' Insight: Discrimination against Indigenous Peoples through the Eyes of Health Care Professionals.  Wylie L, McConkey S | 2019 | Canada | To examine the perspectives of healthcare professionals in order to identify challenges facing indigenous patients in healthcare. | n = 25 participants  (21 individual interviews and 2 focus group discussions). | -Participants reported that healthcare is an unwelcoming environment for indigenous patients  -Views on indigenous people in healthcare were based on negative stereotypes and bias.  -Healthcare professionals reported that racism informs interactions with indigenous people who are often blamed for their health status. |
| How social welfare and health professionals understand “Race,” racism, and whiteness: A social justice approach to grounded theory  Vanidestine, T., & Aparicio, E. M | 2019 | USA | To investigate how social welfare and health professionals understood racial concepts within health disparities discourse. | n =15 healthcare and social care professionals who have experience in teaching. | -The analysis revealed four theoretical categories that characterized how participants understand race, racism and whiteness namely self-defined skin color; (pre)judgments and discrimination; privilege and power; and conceptual conflation and unfamiliarity.  -More emphasis in training healthcare professionals is needed in regards to concepts such as power, inequities and structural racism that moves beyond individual interactions. |
| Racialized Risk in  Clinical Care: Clinician Vigilance and Patient Responsibility  Bell HS, Odumosu F, Martinez-Hume AC, Howard HA, Hunt LM | 2019 | USA | To explore how racialized risk is conceptualized and how it impacts patient care and experience. | n =52 patients. N=19 healthcare professionals. N=122 clinical observations.  n =51 clinic’s medical records | -Most healthcare professionals stated that the patient’s race affected how they cared for the patient. The reasons that were given were biological factors, which the professionals attributed to race e.g. having a higher risk of kidney disease, diabetes etc.  -Most healthcare professionals stated that patients’ racial identity was the same as genetic risk and was used to invoke stereotypes about patients as well as used to justify racialization. |
| Conflict, complicity, and challenges: Reflections on the South African truth and reconciliation commission health sector hearing  Wildschut, G., & Mayers, P. M | 2019 | South Africa | To examine the healthcare and medical violations committed during the apartheid era in South Africa. | Health sectoral hearings from the South African truth and reconciliation commission. | -Healthcare providers committed racial violations including being complicit in torture of antiapartheid activists.  -Due to the complicity of the healthcare sector in racial injustices, training in human rights, empathy was recommended. |
| Case study of a decolonising Aboriginal community controlled comprehensive  primary health care response to alcohol-related harm  Freeman T, Baum F, Mackean T, Ziersch A, Sherwood J, Edwards T, Boffa J. | 2019 | Australia | To examine how health service implement  a community controlled comprehensive  primary health care response to alcohol-  related harm and to examine how this this comprehensive approach challenge the logic and processes of  ongoing colonisation in responses to Aboriginal and Torres Strait Islander  alcohol-related harm. | n =29 interviews with staff members. N=2 congress staffs. N=3 members of partner organisations. N=1 community members.  Service reports from 2009-2013  Community assessment workshops with 13 community members. | -Participants reported both institutional and interpersonal racism and colonialism as key challenges surrounding approaches to alcohol-related harm.  -Participants viewed the government as racist and uncaring towards Aboriginal people.  -Participants reported frustration that racism was often not named. |
| Understanding competing discourses as a basis for promoting equity in primary health care  Blanchet Garneau A, Browne AJ, Varcoe C | 2019 | Canada | To present findings from a critical analysis of the relations among multiple discourses and healthcare practices within four Canadian primary health care clinics that have an explicit commitment to health equity. | n = 31 interviews with clinic staff members. | -Staff members realized that although they strive for equity, implicit unconscious bias might still prevail.  -Staff members understood the importance of continuously examining ones racial bias in healthcare interactions. |
| ***Qualitative articles examining antiracist training for healthcare professionals (n=5)*** | | | | | |
| **Article title and author/s** | **Year of publication** | **Geographical location** | **Aim of study** | **Methods** | **Key findings in regards to racism in healthcare** |
| Training providers on issues of race and racism improve health care equity  Nelson, S. C., Prasad, S., & Hackman, H. W. | 2015 | USA | -To assess a training module to address race and racism. | n =19 physicians participated in a training module. | -The awareness level of issues of racism increased significantly in all participants.  -The impact of racism on health care in general as well as individuals’ ability to deliver care was felt to have increased in all.  -White participants showed a significant decrease in feeling as effective in caring for patients of color when compared to White patients and they felt less equipped to care for patients of color following the training. |
| The lived experience of teaching about race in cultural nursing education  Holland, A. E. | 2016 | USA | To examine nurses’ perceptions on teaching racism in medical education. | n =10 White nurse teachers. | -Whiteness was a barrier to teach about race and nurses were not prepared to teach about issues on racism in healthcare. |
| ‘Just think of TB and Asians’, that’s all I ever hear”: Medical learners’ views about training to work in an ethnically diverse society  Kai, J., Bridgewater, R., & Spencer, J. | 2018 | UK | To explore medical learners' perceptions and their perceived training needs in relation to cultural and ethnic diversity in healthcare. | n =9 focus groups with 55 medical learners in the UK including 46 medical and students and medical practitioner registrars. | -There was inadequate awareness on the meaning of multicultural care and answers from participants were mostly formulated as difference as in difference between various ethnic groups.  -Participants discussed the racialization of diseases such as TB and Asians and saw it as tokenism.  -Most students felt that they needed more training on multicultural care. |
| Communicating with providers about racial healthcare disparities: The role of providers’ prior beliefs on their receptivity to different narrative frames  Burgess, Diana J; Bokhour, Barbara G; Cunningham, Brooke A ; Do, Tam; Gordon, Howard S; Jones, Dina M; Pope, Charlene; Saha, Somnath; Gollust, Sarah E | 2019 | USA | To explore the role of narratives as a vehicle for raising awareness and engaging providers about the issue of healthcare disparities. | n =53 healthcare providers were provided with different narratives in connection to healthcare disparities. | -Participants accepted the “Provider Success” narratives, where interpersonal barriers involving a patient of color were successfully resolved by the  provider narrator.  -“Persistent Racism” narratives, in which problems faced by the patient of color were more explicitly linked to racism, remained unresolved and were very polarizing. |
| “I Didn't Know What to Say”: Responding to racism, discrimination, and microaggressions with the OWTFD approach  Sotto-Santiago, S., Mac, J., Duncan, F., & Smith, J. | 2020 | USA | To assess an educational intervention programme targeting faculty members on racism and inequalities in health. | n =30 people participated in the workshop.  n =24 completed the pre-and post-workshop survey. | -Pre-workshop survey concluded that the majority of the participants did not discuss issues related to racism and discrimination in their workplace.  -Pre-survey workshop indicated that although workshop attendees did not discuss issues pertaining to racism, they often thought about these issues.  -The post-workshop survey showed that the majority agreed or strongly agreed that their professional work would benefit from attending the workshop.  -Most of the attendees reported being familiar with the cases of racism that were presented during the workshop. |
| ***Quantitative articles examining antiracist training for healthcare professionals (n=7)*** | | | | | |
| **Article title and author/s** | **Year of publication** | **Geographical location** | **Aim of study** | **Methods** | **Key findings in regards to racism in healthcare** |
| Making a difference in medical trainees' attitudes toward Latino patients: A pilot study of an intervention to modify implicit and explicit attitudes  Chapman MV, Hall WJ, Lee K, Colby R, Coyne-Beasley T, et al | 2005 | USA | To assess an intervention in a medical education setting designed to change discriminatory attitudes against Latinos in the USA. | A sequential cohort, post-test design.  n =69 medical trainees at a university hospital.  Two groups were compared: The intervention group n = 41 and the control group n = 28. | -Participants in the intervention group showed greater ethnocultural empathy, healthcare empathy, and patient-centeredness, compared to the comparison group. However, no change in implicit bias towards Latinos was seen between intervention and control groups. |
| Bringing interdisciplinary and multicultural team building to health care education: the downstate team-building initiative  Joanie Mayer Hope, Daniel Lugassy, Rina Meyer, Freida Jeanty, Stephanie Myers, Sadie Jones, Joann Bradley, Rena Mitchell, Eva Cramer | 2005 | USA | -To evaluate the impact of a multicultural and interdisciplinary health  care team-building program for health professions students (Downstate Team-Building Initiative). | n =65 students interdisciplinary  - The intervention (DTBI) was used to discuss the meaning of race and also the norms, customs, and values associated with it. | -Significant change was seen in connection to all variables.  -Confident in addressing racism in healthcare increased post intervention. |
| Using a structural competency framework to teach structural racism in pre-health education  Metzl, J. M., Petty, J., & Olowojoba, O. V. | 2009 | USA | To examine medical students perceptions of structural factors and health outcomes | n =185 medical student major. n =63 premeds.  n =91 first semester freshmen | -All groups showed some understanding of structural racism.  -Students who graduated from an interdisciplinary pre-health curriculum identified relationships between structural factors and health outcomes more than premedicine science majors did. |
| Health Equity Rounds: An Interdisciplinary Case Conference to Address  Implicit Bias and Structural Racism for Faculty and Trainees  Perdomo J, Tolliver D, Hsu H, He Y, Nash KA, Donatelli S, Mateo C, Akagbosu C, Alizadeh F, Power-Hays A, Rainer T, Zheng DJ, Kistin CJ, Vinci RJ, Michelson CD | 2019 | USA | To engage faculty and practitioners across training levels and disciplines in discussions around structural racism and healthcare professionals’ implicit bias.  To evaluate the educational sessions through surveys. | n =A mean of 66 attendees per educational session (6 sessions in total). | -The majority of the survey respondents rated the educational value as good or excellent.  -The majority of the survey respondents indicated that the sessions promoted personal reflection on implicit bias. |
| Impact of a Discussion Series on Race on Medical Student  Perceptions of Bias in Health Care.  Bright HR, Nokes K | 2019 | USA | To examine baseline perceptions of first-year medical students about race and healthcare. | n =25 first year medical students who participated in a discussion on race in healthcare  N=180 non-participants. | -Participants were significantly more likely than non-participants to feel that the medical school curriculum should provide more discussion on race and racism.  -There was a significant higher reported comfort with talking about racism in healthcare. |
| Recognizing Racism in Medicine: A Student-Organized and Community-Engaged Health Professional Conference. Health Equity  Adelekun AA, Beltrán S, Carney J, Lett LA, Orji WU, Rider-Longmaid E, Stokes DC, Teeple S, Aysola J | 2019 | USA | To evaluate the implementation of a student-led intervention designed to train health professionals on the impact of racism in health care and provide tools to mitigate it. | n =220 surveys completed by the intervention participants including medical, nursing and social students. | -47.7% participants reported being more comfortable discussing how racism affects health, 36.4% had better understanding of the impact of racism on an individual’s health and 54.5% felt more connected to other health professionals working to recognize and address racism in medicine. |
| Teaching Intersectionality of Sexual Orientation, Gender Identity, and Race/Ethnicity in a Health Disparities Course  Bi S, Vela MB, Nathan AG, Gunter KE, Cook SC, López FY, Nocon RS, Chin MH | 2020 | USA | To explore a module of teaching on racism in healthcare as well as health disparities among students in a medical institution in the USA | n =83 medical students | -Students felt more confident in definition intersectionality after attending the educational module.  -Students also reported a better understanding of identifying barriers to care for sexual and gender minorities.  -The majority of students rated the session as very good or excellent. |
| ***Mixed method articles examining antiracist training for healthcare professionals (n=3)*** | | | | | |
| **Article title and author/s** | **Year of publication** | **Geographical location** | **Aim of study** | **Methods** | **Key findings in regards to racism in healthcare** |
| Confronting uncomfortable truths: receptivity and resistance to Aboriginal content in midwifery education  Thackrah, R. D., & Thompson, S. C | 2018 | Australia | -To explore the views of midwifery students on compulsory Aboriginal health unit. | Quantitative study: before (education) and after questionnaires were obtained. | -Students demonstrated a somewhat changed perceptions towards Aboriginal people as well as challenging stereotypes. However, issues around racism were not resolved. |
| Disruption as opportunity: Impacts of an organizational health equity intervention in primary care clinics  Annette J. Browne, Colleen Varcoe, Marilyn Ford-Gilboe, C. Nadine Wathen, Victoria Smye, Beth E. Jackson, Bruce Wallace, Bernadette (Bernie) Pauly, Carol P. Herbert, Josée G. Lavoie, Sabrina T. Wong & Amelie Blanchet Garneau | 2018 | USA | To evaluate an intervention in healthcare settings aiming at increasing equity in healthcare (effects of structural inequities, impact of racism, discrimination and stigma on people’s access to services and their experience of care and the mismatches between dominant approaches to care and the needs of people most effected by health inequities. | *Quantitative*  Survey collected at three points with healthcare providers  n = 86, n =82, n =57 respectively  *Qualitative*  Observational data (fieldnotes 380 hours) | -The intervention increased awareness and confidence as well as drew attention on previously salient health inequities and social determinants of health such as racism in healthcare at both the individual and organisational challenges.  -The intervention challenged the status quo and in some cases healthcare providers even resisted challenges to health inequities. |
| Exploring cultural safety with Nurse Academics. Research findings suggest time to "step up"  Doran F, Wrigley B, Lewis S | 2019 | Australia | To explore capability in relation to cultural safety (including antiracism in healthcare) with Nurse  Academics at a regional university in New South Wales, Australia. | n =15 staff members completed the online-survey. | -Over 2/3 of the participants reported a good understanding of cultural safety  -Many participants reported interests in antiracist training.  -Participants reported a variation in understanding cultural safety. |
